# Hybrid Supervised‐Unsupervised Modeling for Post‐Hurricane Private Well Contamination Risk Score Using Empirical Validation and Community‐Informed Assessment

**DOI:** 10.1029/2026GH001858

**Published:** 2026-06-17

**Authors:** Jilei Lin, Jennifer Zhang, Ellen Wei, Kyndra Shea, Huixia Judy Wang, Tatiyana V. Apanasovich, Jahred M. Liddie, Erika Hernandez, Crystal Norford, Xindi C. Hu

**Affiliations:** ^1^ Department of Statistics The George Washington University Washington DC USA; ^2^ Department of Environmental and Occupational Health The George Washington University Washington DC USA; ^3^ Thomas Jefferson High School for Science and Technology Alexandria VA USA; ^4^ Department of Statistics Rice University Houston TX USA; ^5^ Clean Water for North Carolina Stem NC USA

**Keywords:** geospatial modeling, ground truthing, well water, hurricane helene, microbial contamination

## Abstract

Hurricane‐related flooding can mobilize microbial and chemical contaminants, while limited well testing and uneven disaster response capacity leave many households relying on private wells at elevated risk, particularly during the early recovery phase when contamination risk is acute. To address this challenge, we propose a data‐driven framework for quantifying post‐hurricane private well contamination risk. The framework produces a numerical risk score by integrating 78 geospatial variables across three modules representing hazard, physical vulnerability, and social capacity. The score is constructed using a hybrid approach that combines supervised and unsupervised learning to generate interpretable indices grounded in theory and calibrated to real‐world data. We applied the framework to western North Carolina following Hurricane Helene and evaluated its performance using post‐hurricane well testing data from the North Carolina Department of Health and Human Services together with community‐informed assessments in two counties. Higher risk scores were significantly associated with increased total coliform contamination (*p* = 0.006, Wilcoxon rank‐sum test), demonstrating the value of the framework for identifying areas with elevated contamination risk. These findings suggest that the framework can help identify areas with elevated contamination risk following extreme weather events, although predictive performance remains moderate and further systematic evaluation is needed. The framework is designed to be transferable and can be adapted to other storms and regions where geospatial and well testing data are available. Overall, this work provides a practical, data‐informed tool to support disaster preparedness, prioritize well testing, and protect private well users after extreme weather events.

## Plain Language Summary

Millions of people in the United States rely on private wells for drinking water, especially in rural areas. Unlike public water systems, private wells are not regulated, so contamination often goes unnoticed unless homeowners test their water. After hurricanes and flooding, the risk of contamination increases because floodwaters can carry bacteria and chemicals into wells. At the same time, testing resources are limited, making it hard for health agencies to know where help is most urgently needed. In this study, we developed a data‐driven approach to estimate the risk of private well contamination after hurricanes. The method creates a single risk score by combining information on flooding, well and land characteristics, and community resources that may reduce risk. We applied this approach in western North Carolina after Hurricane Helene and evaluated it using well water test results and field observations in local communities. We found that wells with higher risk scores were more likely to have bacterial contamination. This suggests the framework can help identify areas where testing and assistance should be prioritized. Although demonstrated for one hurricane, the approach can be used in other regions and storms to support disaster preparedness and protect private well users after extreme weather.

## Key Points

- Contamination risk scores guide where to test private wells first, especially after hurricanes and flooding
- We use a hybrid framework combining supervised and unsupervised learning to build interpretable and empirically validated risk scores
- Community‐informed assessment informs and calibrates model construction, using local knowledge to guide targeted well testing and outreach

## 1 Introduction

Over 43 million U.S. residents (13% of the population), many in rural or underserved areas, rely on private wells for drinking water (Dieter et al., 2018). These systems are not regulated under the Safe Drinking Water Act, which applies only to public water systems serving at least 15 service connections or 25 individuals (U.S. Environmental Protection Agency, 2023). Because private wells are unregulated by the federal government, water contamination can often remain undetected unless well users conduct water testing independently. In North Carolina, only 6% of the predicted 1.6 million wells have been tested since February 2009, with barriers such as cost, inconvenience, mistrust, optimism bias, and limited awareness reducing testing uptake (Hayes et al., 2025).

Extreme weather further compounds these vulnerabilities. Hurricane flooding elevates microbial contamination in well water, contributing to gastrointestinal illness and adverse birth outcomes (Hochard et al., 2022; Quist et al., 2022). With climate change driving more intense category 4 and 5 storms, risks are projected to grow (Knutson, 2024; Salarieh et al., 2023). Hurricane Helene made landfall in Florida in September 2024 and subsequently tracked through western North Carolina as a tropical/post‐tropical system, causing $59.6 billion in damages, over 100 deaths, and widespread loss of water, power, and healthcare access (Cooper, 2024). Helene's effects were concentrated in 25 western counties, a rural and mountainous region where many residents rely on private wells. This forms the basis of our case study. In response, the North Carolina Department of Health and Human Services (NC DHHS) offered free well testing and disinfection kits to affected households (North Carolina Department of Health and Human Services, Division of Public Health, 2024). Prior events show that flooding threatens the safety of private wells by mobilizing microbial and chemical contaminants such as *Escherichia coli* (*E. coli*), total coliform, and nitrate (Naylor et al., 2018). For example, after Hurricane Harvey, *E. coli* detection rates in wells were nearly three times higher than baseline (Pieper et al., 2021).

These challenges highlight an urgent need for a framework to help residents understand their likelihood of well water contamination and guide targeted public health interventions in the aftermaths of hurricanes and other flooding events. Similar challenges have been widely documented in environmental pollution studies, including dust and heavy metal contamination in industrial and mining regions, where multivariate and statistical approaches are used to characterize pollutant sources, exposure pathways, and associated health risks (Barjoee et al., 2020, 2024; Nazari Alamdarloo et al., 2020; Shojaee Barjoee et al., 2020). Drawing on recent advances in flood‐risk assessment, particularly the Integrated Risk Linkages framework introduced by (Tabasi et al., 2025), we conceptualize post‐hurricane private‐well contamination as a risk emerging from the intersection of hazard, physical vulnerability, and social capacity. In this conceptual framework, hazard (the intensity and extent of flooding) interacts with system‐specific susceptibility (here, well construction, hydrogeology, and landscape characteristics) to increase the risk, while social capacity reduces the risk through access to resources, information, and adaptive actions. Yet despite the relevance of this integrated framing, no existing index comprehensively operationalizes all three components for private‐well contamination, particularly in post‐hurricane settings where timely, actionable risk characterization is critical.

Existing index‐building approaches fall broadly into unsupervised and supervised categories, each with notable trade‐offs. Unsupervised, theory‐driven approaches, such as the method used in constructing the CDC Social Vulnerability Index (Centers for Disease Control and Prevention et al., 2024), rely on expert knowledge to weight variables and are broadly generalizable. However, they often lack empirical validation and community ground‐truthing. They can also be highly sensitive to analytical choices such as variable selection, dimensionality reduction method, and spatial scale (Hinojos, 2025). In contrast, supervised, data‐driven approaches rely on observed data to train predictive models, including machine learning models such as random forest, XGBoost, support vector machines, and decision trees. These models can perform well when high‐quality and representative data are available. However, environmental data are often sparse or noisy, which limits predictive power and can embed existing biases, reducing generalizability to new situations. The individual limitations of unsupervised and supervised methods motivate a hybrid approach that integrates their respective strengths.

In this study, we develop a hybrid and statistically principled framework for post hurricane private well contamination risk assessment. We integrate dimension reduction techniques with supervised learning to construct concise and interpretable indices for three modules: hazard, physical vulnerability, and social capacity. Using empirical post hurricane well testing data, we assess the effectiveness of the resulting composite risk score in identifying areas with elevated contamination risk. We also conduct community informed assessments with partners in North Carolina to qualitatively assess the social capacity module. Although demonstrated in a specific post hurricane context, the proposed framework is designed to be transferable and can be applied to other hurricanes, regions, and broader disaster related settings where geospatial data and well testing information are available, supporting more equitable allocation of well testing resources.

## 2 Methods

### 2.1 Post‐Hurricane Private Well Contamination Risk Conceptual Framework

**Figure 1:**
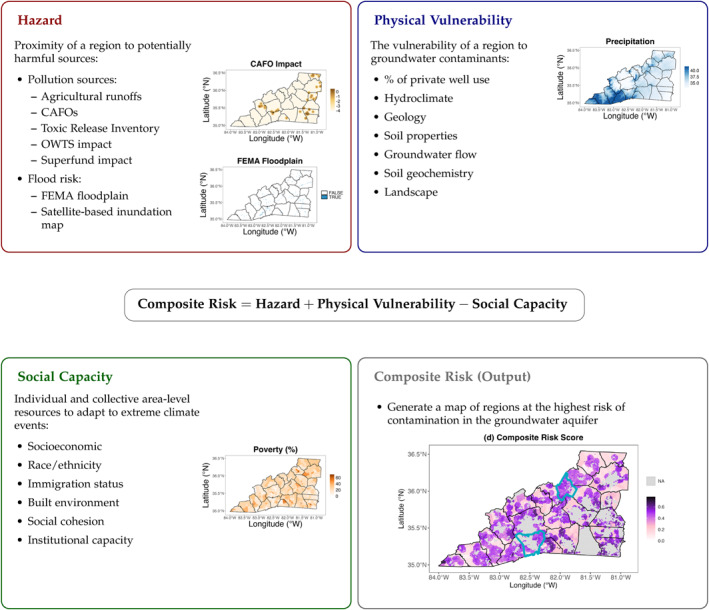
Post‐Hurricane Helene private well contamination risk conceptual framework in 25 western counties of North Carolina, USA. The framework integrates three modules: hazard, physical vulnerability, and social capacity, each capturing distinct sets of variables (Table S1 contains the full list). The boundaries of Avery and Henderson Counties, where we conducted ground‐truthing, are emphasized with bold, teal outlines in the lower‐right panel. The composite risk score is derived as a function of the indices from these three modules, based on the formula shown in the diagram. Abbreviations: OWTS = Onsite Wastewater Treatment Systems; FEMA = Federal Emergency Management Agency; CAFOs = Concentrated Animal Feeding Operations.

The post‐hurricane private well contamination risk framework comprises three modules: hazard, physical vulnerability, and social capacity (Figure 1). The hazard module represents potential contamination sources and flood exposure. Physical vulnerability captures the susceptibility of groundwater to contamination based on local soil, geologic, and hydrologic characteristics. Social capacity reflects a community's ability to mobilize resources for adaptation and recovery. We construct an index for each module independently and then combine them using a simple and intuitive additive formula to obtain a composite risk score, in which social capacity reduces risk while hazard and physical vulnerability increase it. Our finding is robust to alternative formulations of the composite risk score; in a sensitivity analysis using a multiplicative specification (Hazard × Vulnerability × 1/Capacity). Details are provided in Section S4 and Table S8 of the Supporting Information S1.

### 2.2 Variables Incorporated in Each Risk Module

In Sections 2.2.1, 2.2.2, and 2.2.3, we describe the variables incorporated into the hazard, physical vulnerability, and social capacity modules, along with their corresponding data sources. We also explain the conceptual rationale underlying each module. Summary statistics for the variables in each module are provided in Tables S2–S6 in Supporting Information S1. We used *R* version 4.5.1 to conduct all spatial processing and statistical analyses (R Core Team, 2020).

#### 2.2.1 Hazard

Hazard refers to a phenomenon, substance, human activity, or condition that can result in harm, damage, or danger (Pistrika & Tsakiris, 2007; United Nations International Strategy for Disaster Reduction, 2004). In the context of flood risk assessment, hazard encompasses both the probability of flood inundation and the presence of contaminant sources (Table S1). We characterized event‐specific flood inundation using satellite‐derived flood depth estimates from the Global Flood Monitoring System, which combines hydrologic modeling with satellite precipitation inputs (University of Maryland, 2025). We extracted the maximum modeled inundation depth (in millimeters) during Hurricane Helene (24–28 September 2024) to represent flood severity. To capture chronic flood susceptibility, we incorporated US Federal Emergency Management Agency (FEMA) 100‐year floodplain boundaries from the North Carolina Floodplain Mapping Program (North Carolina Flood Risk Information System (FRIS), n.d.). Together, these two variables reflect both acute and long‐term flood exposure.

We integrated multiple data sets to represent chemical and microbial contamination sources that may be mobilized and transported during flood events. Industrial and hazardous waste facilities with potential chemical releases were identified using the EPA Toxics Release Inventory (U.S. Environmental Protection Agency/TRI Program, 2025) and Superfund Enterprise Management System (US EPA Office of Mission Support, 2025). We captured agricultural nutrient loading through crop‐specific nitrogen application rates from the USDA Cropland Data Layer (U.S. Department of Agriculture, National Agricultural Statistics Service, 2020) and Agricultural Chemical Use Program (U.S. Department of Agriculture, 2019), and we represented microbial and nutrient contributions from livestock operations using North Carolina Department of Environmental Quality (NCDEQ) data on concentrated animal feeding operations (North Carolina Department of Environmental Quality, 2024). Human waste inputs were captured using NCDEQ National Pollutant Discharge Elimination System (North Carolina Department of Environmental Quality/Water Quality Permitting Division, 2025) data and county‐level septic system density estimates.

To quantify the influence of these sources, we calculated an impact score using inverse‐distance‐like exponential decay (wi = 1/exp (di/1000)), where di is the Euclidean distance (meters) from each grid cell centroid to source points in projected CRS (EPSG:3358). Sources beyond 5 km (di > 5,000 m) were assigned zero weight, consistent with the distance threshold used in EPA's EJSCREEN proximity score methodology (U.S. Environmental Protection Agency, 2015). Weights were summed across all sources within this range. The decay function is well‐defined at di = 0, so no special handling is required when a source falls within or very close to a grid cell centroid (Pieper et al., 2021). All hazard variables were aggregated to a 1 km × 1 km spatial grid prior to analysis.

#### 2.2.2 Physical Vulnerability

Physical vulnerability captures the physical and environmental susceptibility of an area to the negative impact of flooding (Admin, 2017). We grouped the physical vulnerability variables into the following categories: geology, groundwater, hydroclimate, landscape, soil properties, redox conditions, and domestic well use (Table S1). We selected variables and designated their categories based on prior studies evaluating groundwater contamination (Hynds et al., 2012; Naylor et al., 2018; White et al., 2021) and well vulnerability (Cao et al., 2018; Hochard et al., 2023; Pieper et al., 2021). We also include depth to water table (DeSimone et al., 2009), derived from simulated groundwater conditions, as a proxy for the minimum well depth, given the limited availability of well construction records. All physical vulnerability variables were extracted to the 1 km × 1 km grid described in Section 2.2.1.

Our physical vulnerability module is conceptually related to established groundwater vulnerability indices such as DRASTIC (Aller et al., 1987), as it incorporates several similar hydrogeologic and environmental dimensions. For example, depth to water table correspond directly to the “D” (depth to groundwater) component; precipitation‐ and runoff‐based indicators serve as proxies for “R” (net recharge); aquifer characteristics such as lithology and permeability align with “A” (aquifer media); soil texture and drainage properties correspond to “S” (soil media); terrain slope and elevation‐derived measures reflect “T” (topography); and subsurface conditions such as redox‐related variables provide information related to “I” (impact of the vadose zone) and “C” (hydraulic conductivity).

However, rather than relying on expert‐defined ratings and weights, we combine these variables using a data‐adaptive hybrid supervised–unsupervised framework. We also extend beyond traditional indices by incorporating predictors specifically relevant to post‐hurricane total coliform contamination risk, including hydroclimate indicators, redox‐related variables, land use characteristics, and domestic well use.

To estimate domestic well use, we combined census block‐level well use estimates from Murray et al. (2021) with 2020 TIGER/Line census block geometries. We spatially joined the centroids of all grid cells with their overlapping census block groups to assign well‐use estimates. For vector data sets (polygons) describing geology, groundwater, hydroclimate, and soil properties, we assigned attribute values to each grid cell based on the centroid (Table S1). For raster data sets, we used the *exactextractr R* package to compute zonal statistics over the 1 km by 1 km grid, after aligning all coordinate reference systems before joining.

For integrating land use characteristics (e.g., elevation, % agricultural land, % impervious land), we first selected variables from the US EPA EnviroAtlas database (Pickard et al., 2015), which are available at 12‐digit hydrologic unit code, and then similarly performed centroid‐based spatial joins. We incorporated raster‐based redox‐related predictors using machine learning predictions of elevated manganese and oxic conditions to capture additional risks to contaminant transport and toxicity (Tesoriero et al., 2024). When missing values were present in these data, we used inverse distance weighting interpolation to impute concentrations at a 5 km resolution with a 60 km buffer around North Carolina (U.S. Environmental Protection Agency, 2015).

For other variables with missingness, we applied a centroid‐based spatial k‐nearest neighbors approach, where each grid cell with missing data was imputed using its five nearest neighboring cells with observed values; continuous variables were imputed using the median of neighboring values, and categorical variables using the most frequent category (mode). We note that alternative approaches based on model‐driven estimation and simulation have also been used in environmental risk analyses to address incomplete data (Shojaee Barjoee, Azizi, Kouhkan, et al., 2023), although such methods were not implemented in the present study because the large number of variables would require a dedicated investigation of the underlying missing‐data mechanisms and model specification.

#### 2.2.3 Social Capacity

Social capacity describes the ability of a community to prepare for, respond to, and recover from hurricanes, natural disasters, and other major disturbances. It encompasses demographics, socioeconomic status, health risks, immigration status, social cohesion, institutional capacity, the built environment, and other social determinants. Communities with high social capacity can respond more quickly and effectively to disturbances, whereas communities with low social capacity face greater challenges in recovery and resilience.

Established indices informed our variable selection. The CDC Social Vulnerability Index (Centers for Disease Control and Prevention et al., 2024) includes measures such as minority status, limited English proficiency, unemployment, educational attainment, poverty, health insurance coverage, and vehicle ownership. The Area Deprivation Index (Kind & Buckingham, 2018) incorporates housing and infrastructure measures, including homeownership, plumbing, and internet access. Although these variables are commonly used to characterize social vulnerability, in our framework they are reoriented to represent adaptive capacity by aligning their directions so that higher values consistently indicate greater capacity (e.g., reverse‐coding where appropriate). Based on input from conversations with local health department officials, we also included the percentage of government workers as a proxy for institutional capacity, and home age as a variable for whether wells were constructed under modern standards (fully implemented in 2008) (North Carolina General Assembly, 2024).

All social capacity variables were obtained from the 2019–2023 American Community Survey 5‐year estimates, reported as percentages based on the relevant total population (U.S. Census Bureau, 2023). We used the finest available spatial resolution, the block group level, except for variables only reported at the census tract level (Table S1). Values for each block group or census tract were then mapped onto the 1 km × 1 km grid, with each grid cell assigned the value of the block group or census tract containing its centroid, as described in Section 2.2.2. Missing values were handled using a similar spatially informed approach as in the physical vulnerability module, where imputation was based on neighboring grid cells identified through centroid‐based proximity.

### 2.3 NC DHHS Well Testing Data Set

To calibrate our risk score using real‐world contamination data, we extracted total coliform test records from the North Carolina State Laboratory of Public Health (NC Department of Health and Human Services ‐ Environmental Public Health Tracking Program, 2025) using web scraping (Richardson, 2025; Singer‐Vine, 2025), parsed PDF lab reports, and geocoded street addresses using Geocod.io (Geocodio, 2025) (*n* = 7,726). Following Hurricane Helene, both hurricane‐specific and routine statewide tests were available. We excluded samples containing treated water (*n* = 481, 168 hurricane‐specific, 313 routine), records with insufficient address information (*n* = 12), and agricultural samples (*n* = 237). We merged hurricane‐specific and routine statewide data sets, removed duplicates, and retained the test collected closest to the end of the hurricane for sites sampled multiple times (*n* = 4,337). Results were reported as “Absent”/“Present” or as most probable number/100 mL.

To focus on regions affected by Hurricane Helene, we restricted analyses to samples collected from 25 western North Carolina counties (*n* = 1,159). Because contamination levels declined sharply by March 2025, approximately five months after the hurricane and consistent with prior studies (Pieper et al., 2021), we further limited analyses to samples collected before 1 March 2025 to capture the period of elevated post‐hurricane risk (*n* = 623).

In this data set, cases are defined as wells with detected contamination among submitted samples, and controls are submitted samples with non‐detect results. Accordingly, the outcome reflects detected contamination conditional on testing sample submission, rather than population‐level contamination prevalence across all private wells. Each well was mapped to its corresponding grid cell and assigned all variable information from the hazard, physical vulnerability, and social capacity modules, along with the derived indices and composite risk scores (as described in Section 2.4). These data are used for model calibration and internal evaluation of the risk score, rather than external validation.

### 2.4 Hybrid Supervised‐Unsupervised Approach for Index Construction

We developed a hybrid supervised–unsupervised framework to synthesize variables from the hazard, physical vulnerability, and social capacity modules into a composite post‐hurricane contamination risk score. The core idea of this approach is consistent with a growing body of literature that combines unsupervised dimension reduction with supervised outcome‐guided refinement (Bair et al., 2006; Sugiyama, 2007; Zheng et al., 2025). Specifically, these methods typically (a) apply unsupervised learning techniques, such as principal component analysis (PCA), to reduce the dimensionality of covariates; (b) use labeled outcome data in a supervised model (e.g., logistic regression) to refine or reweight the reduced features; and (c) construct a single composite index using the supervised model–derived coefficients. Variants of this strategy have been applied across diverse domains, including risk prediction and disease modeling (Zheng et al., 2025), with differences primarily arising from the choice of models used in the unsupervised and supervised stages.

**Figure 2:**
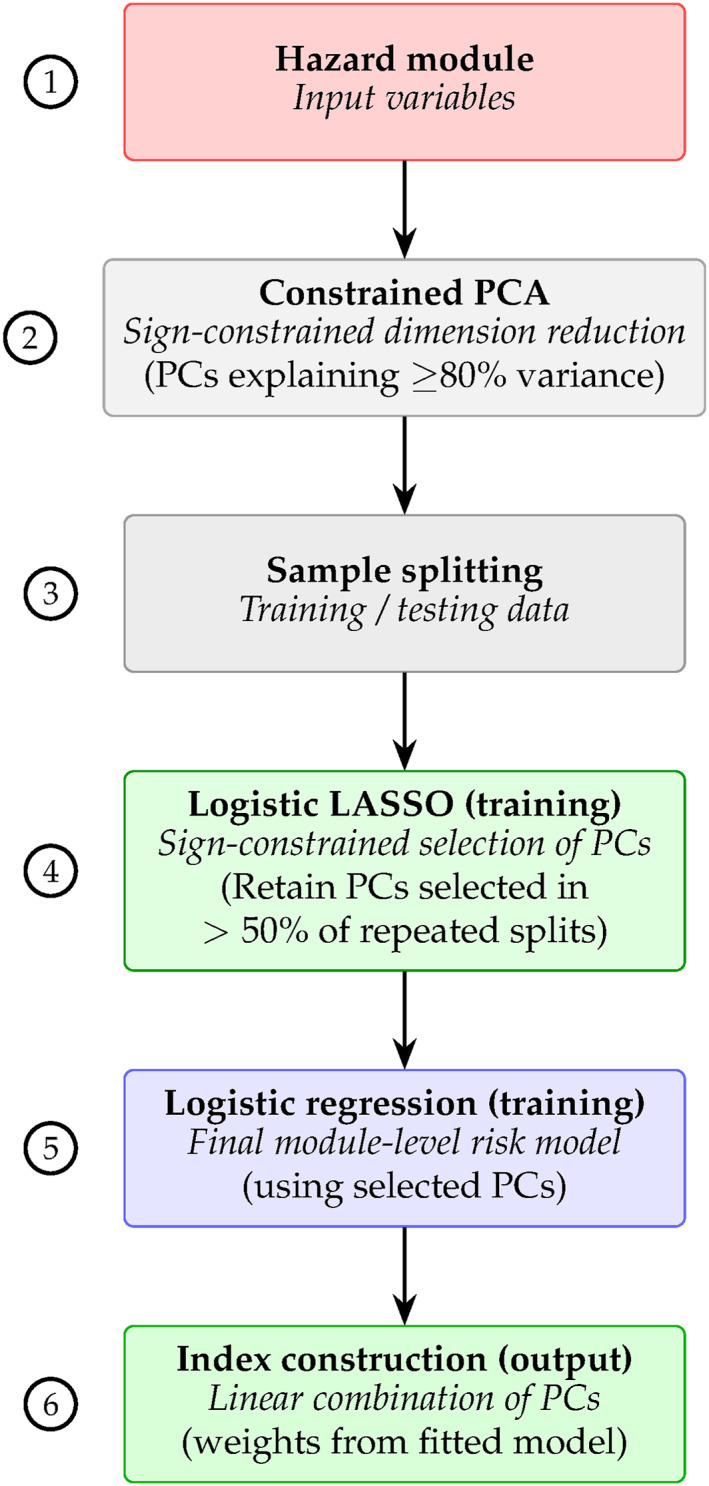
Example flowchart of the hybrid supervised–unsupervised framework used to construct the hazard index. The hazard index is one of three indices (in addition to physical vulnerability and social capacity) used to develop the composite risk score. PCA: principal component analysis; PC: principal component; LASSO: least absolute shrinkage and selection operator.

Figure 2 illustrates the workflow for constructing the hazard index using this hybrid framework; the same procedure is applied to the other modules. Below, we outline the key steps, with full implementation details provided in Section S3 in Supporting Information S1.

We first applied sign‐constrained PCA separately to the correlation matrix of each module (hazard, physical vulnerability, and social capacity) to obtain an informative, lower‐dimensional set of principal components (PCs). In this context, weights refer to the coefficients used to compute each PC score as a linear combination of the original variables, whereas loadings describe the strength of association between each original variable and a given PC.

To improve interpretability and align with the conceptual direction of each module, we imposed sign constraints on selected coefficients based on subject‐matter expectations (e.g., variables associated with greater hazard or vulnerability were constrained to have coefficients in the same direction, whereas social capacity variables were constrained in the opposite direction). This yields a constrained eigenvector optimization problem rather than a post hoc rotation or a sparse PCA variant. Although such constraints may reduce the variance explained relative to ordinary PCA, we found the loss to be modest across modules. Details of the formulation, variance comparison, and variable‐specific sign assignments are provided in Section S3.1 of Supporting Information S1 (Table S1 and Figure S1 in Supporting Information S1). We also refer readers to Barjoee et al. (2024) for an applied example of PCA‐related methods.

To mitigate the influence of outliers on numerical stability and estimation, we centered and standardized the variables (see details in Section S1 in Supporting Information S1). The imposed sign constraints were applied to the PC weights, ensuring that the directions of influence aligned with subject‐matter expectations (see Table S1). Within each module, we retained the PCs that jointly explained at least 80% of the total variance, yielding a reduced yet informative set of features.

Next, we randomly split the NC well‐testing data set into training (*n* = 374) and testing (*n* = 249) subsets. Training data were used both to calibrate the module‐specific index via sign‐constrained logistic regression and to train random forest models for evaluating the effectiveness of the dimensionality reduction. Testing data were reserved for out‐of‐sample evaluation, including validation of the composite risk score. For the probabilistic classification models, we chose a probability threshold that improves detection of contaminated wells at the cost of more false positives, which is more consistent with the intended use of the score as a screening and prioritization tool.

We acknowledge that a spatial cross‐validation scheme, such as leave‐one‐county‐out or spatial block cross‐validation, would provide a more rigorous assessment of predictive performance. However, because the wells are unevenly distributed across only 22 counties, with several counties contributing very few observations, such spatial partitioning would be unstable in the current data set.

Because the number of retained PCs was large, we split the validation data into training and testing sets using a stratified 60/40 split to balance stable model estimation with maintaining a sufficiently large test set for statistical comparison. Using smaller test sets (e.g., 70/30 or 80/20 splits) reduces the power of the two‐sample comparison used for evaluation. We then applied module‐specific logistic regression with a LASSO penalty (Tibshirani, 1996) to the training data to identify the most informative PCs for predicting contamination. The LASSO penalty promotes sparsity by shrinking noninformative coefficients toward zero. To ensure interpretability, we constrained the signs of the regression coefficients to align with expected directions of influence based on subject‐matter knowledge. The same sign constraints used in the sign‐constrained PCA were applied here (see Table S1 “sign constraint” column).

The tuning parameter was selected via 10‐fold cross‐validation using area under curve (AUC) for a receiver operating characteristic (ROC) curve as the performance criterion. From the cross‐validated solution path, we chose the penalty value that yielded a model with approximately five nonzero coefficients (active PCs) per module. To reduce sensitivity to any single train‐test split, we repeated the logistic LASSO procedure 500 times, each using an independent random split and retained only PCs that were selected in more than 50% of the iterations through majority voting. A sensitivity analysis of this 50% threshold is provided in Section S3.2 in Supporting Information S1.

Using the selected PCs within each module, we constructed module‐specific indices by forming weighted sums of these PCs, with the coefficients obtained from sign‐constrained logistic regression fitted on the training data. Both the LASSO procedure and the sign‐constrained logistic regression were implemented in *R* using the package *glmnet* (Friedman et al., 2024).

As a robustness check, we assessed residual spatial autocorrelation in the sign‐constrained logistic regression models using Moran's I computed from the Pearson residuals, which has been widely used to characterize spatial dependence in environmental contamination data (Shojaee Barjoee, Azizi, Khaledi, et al., 2023). This analysis was implemented using the *spdep* package (Bivand, 2022) with a 10‐nearest‐neighbor spatial weights matrix constructed from the well coordinates.

### 2.5 Construction and Evaluation of the Composite Risk Score

We combined the module‐specific indices into a composite risk score following the formula in Figure 1 and evaluated its predictive performance on the testing data set. To evaluate the effectiveness of dimension reduction, we trained three random forest models: (a) the full set of PCs (“hybrid” approach); (b) all original variables from the three modules (mimicking the “supervised” kitchen‐sink approach); and (c) only the first PC from each module (mimicking the “unsupervised” approach). Model performances were compared using the AUC metric, along with confidence intervals estimated using DeLong's nonparametric method (DeLong et al., 1988).

Finally, to assess how well the constructed risk score differentiates between contaminated and uncontaminated wells, we applied the Wilcoxon rank‐sum test on the held‐out testing data, using a one‐sided alternative and a significance level of 0.05. The test was performed using the *stats* (R Core Team, 2020) package in *R*.

### 2.6 Community‐Informed Assessment and Outreach

We selected two western North Carolina counties (Avery and Henderson) for the community‐informed assessment and outreach in August 2025 (Figure 1), after contacting all 25 environmental health departments from western North Carolina counties through multiple phone calls and emails. Selection was based on the expressed interest of the Environmental Health Director, the number of post‐Helene well samples reported, and the severity of local flooding impacts. Within each county, we chose outreach areas using spatial maps of the social capacity index (Section 2.2.3), municipal water utility maps, and direct consultation with Environmental Health Directors. Consistent with Clean Water for North Carolina (CWFNC)'s Environmental Justice mission (Clean Water for North Carolina, 2023), we prioritized communities with low social capacity. Outreach areas were therefore not selected as a random sample; rather, this targeted design was intended to assess the index's ability to identify vulnerable communities and does not provide a comprehensive or generalizable validation of its spatial distribution.

We note that field activities were conducted approximately 11 months after Hurricane Helene; while some short‐term flood impacts may have changed, the assessment primarily reflects underlying social capacity and longer‐term recovery conditions, which are less sensitive to this time lag.

To qualitatively assess and refine these selections, the CWFNC team conducted windshield surveys in areas with significant flood damage and a high concentration of private wells, documenting flood impacts and housing conditions as proxies for socioeconomic status. Spatial maps of the social capacity index were provided to the field team, and field observations were used to evaluate whether the smoothed, grid‐based index aligned with on‐the‐ground conditions and to identify local heterogeneity or contextual factors not captured in the available covariates. Door‐to‐door outreach was conducted along selected roads to distribute information on the availability of free bacterial well water testing and to provide interested households with sterile sampling vials and instructions for collection and return to county health departments.

This component is intended to assess and refine the social capacity module and does not constitute validation of the composite contamination risk score, which is evaluated separately using contamination outcomes.

During the preparation of this manuscript, generative AI tools (ChatGPT, developed by OpenAI) were used solely to assist with language refinement and code presentation. All scientific content, analyses, and interpretations were developed and verified by the authors. The authors reviewed and approved the final manuscript and assume full responsibility for its accuracy, integrity, and originality.

## 3 Results

### 3.1 Model Comparison

**Figure 3:**
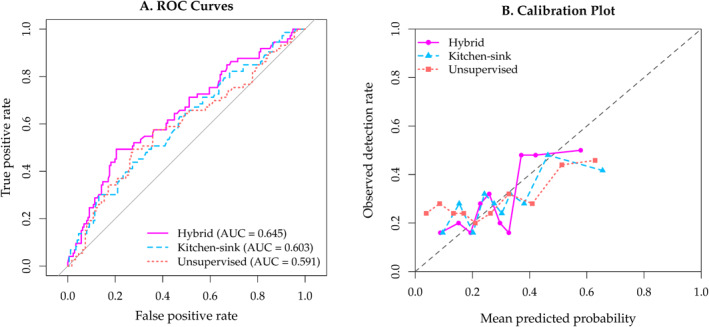
Receiver operating characteristics (ROC) curves and calibration plots for the three random forest models. The hybrid model is trained on the full set of principal components (PCs), the kitchen‐sink model uses all original variables from the three modules, and the unsupervised model relies only on the first PC from each module. Panel A shows test‐set ROC curves, and Panel B shows calibration plots, where the diagonal line denotes perfect agreement between predicted probabilities and observed detection rates.

Hybrid model outperforms kitchen‐sink and unsupervised models across multiple metrics, used for evaluating the performance of classifiers (Table 1). The visual patterns in Figure 3 are consistent with these numerical results. In Panel A, the ROC curve for the hybrid model lies above those of the kitchen‐sink and unsupervised models across much of the threshold range, indicating near‐uniform dominance in discrimination performance. This suggests that the hybrid approach improves discrimination relative to both alternatives, although the gains are modest and not statistically significant. In Panel B, the hybrid model more closely follows the diagonal reference line, indicating better calibration, and it also achieves the lowest Brier score, reflecting more accurate probability predictions. Although overall discrimination remains modest, the hybrid approach provides the strongest combined performance in terms of both ranking ability and probability calibration. Taken together, these results suggest that the hybrid approach preserves much of the predictive signal in the original variables while offering a more parsimonious framework for risk‐score construction.

**Table 1:** Predictive Performance of the Hybrid, Kitchen‐Sink, and Unsupervised Models on the Testing Set

Model	AUC (95% CI)	Sensitivity	Specificity	PPV	NPV	BS
Hybrid	0.645 (0.568–0.721)	0.630	0.540	0.362	0.779	0.197
Kitchen‐sink	0.603 (0.525–0.681)	0.644	0.500	0.348	0.772	0.207
Unsupervised	0.591 (0.510–0.671)	0.603	0.523	0.344	0.760	0.216

*Note.* AUC = area under the receiver operating characteristic curve; CI = confidence interval; PPV = positive predictive value; NPV = negative predictive value; sensitivity = true‐positive rate; specificity = true‐negative rate; BS = Brier score (mean squared difference between predicted probabilities and observed outcomes). Threshold‐based metrics are reported at the training‐derived cutoff selected to maximize the F3‐score.

The hybrid model achieved a sensitivity of 0.630 and a specificity of 0.540. Here, sensitivity is the true positive rate and specificity is the true negative rate; positive predictive value is the proportion of wells predicted to be contaminated that were actually contaminated, and negative predictive value is the proportion of wells predicted to be uncontaminated that were actually uncontaminated. The relatively higher sensitivity indicates that the model is more effective at identifying contaminated wells than avoiding false positives, which is desirable in a screening context where missing contaminated wells may carry greater public health risks.

### 3.2 LASSO‐Informed Index Construction

Table 2 summarizes the PCs selected by the logistic LASSO via majority voting and the high‐weight variables associated with each PC. Within the hazard module, principal components PC5 and PC6 were selected. Inspection of the constrained PCA weights showed that PC5 placed all of its weight on the FEMA floodplain variable (weight = 1.00), with all other hazard variables receiving weights of 0.00 on this component. PC6 placed all of its weight on onsite wastewater treatment systems (weight = 1.00), with all other variables receiving weights of 0.00. Thus, PC5 captures spatial variation in floodplain exposure, whereas PC6 reflects potential contamination sources associated with household wastewater disposal (Gyimah et al., 2024). The event‐specific flood inundation variable was not retained among the selected principal components. In the subsequent logistic regression, both PC5 and PC6 received positive coefficients, with magnitudes in an approximate ratio of 10 to 8.

**Table 2:** High‐Weight Variables for the PCs Selected via Majority Voting From the Logistic LASSO for the Physical Vulnerability, Social Capacity, and Hazard Modules

Module	PC	High‐weight variables	Coefficients
Physical Vulnerability	PC 1	Depth to water table; Transmissivity; Mean annual temperature; % wetlands; % forest; Elevation; Thermic soil temperature regime	0.104
	PC 10	Transmissivity; Stream density; Available water capacity; Soil depth; Bedrock type	0.074
Social Capacity	PC 2	Seniors; Plumbing; Home age	−0.012
	PC 4	Government jobs	−0.078
	PC 8	Plumbing	−0.354
	PC 10	No internet	−0.124
Hazard	PC 5	FEMA Floodplain	0.100
	PC 6	Onsite wastewater treatment systems	0.083

*Note.* High‐weight variables are defined as those with absolute weights greater than 0.25. Within each module, the reported coefficients are the estimated coefficients from the sign‐constrained logistic regression of detect versus non‐detect outcomes on the retained PCs, and they determine the contribution of each selected PC to the corresponding module‐specific index.

Within the physical vulnerability module, principal components PC 1 and PC 10 were selected. PC 1 appears to represent a broad gradient combining hydrogeologic and landscape characteristics, with higher values associated with shallower water tables, higher transmissivity, wetter or more forested land cover, and warmer soil regimes. PC 10 captures variation in subsurface storage capacity and surface–groundwater connectivity. In the logistic regression model, PC 1 and PC 10 were assigned positive coefficients, with magnitudes in an approximate ratio of 10 to 7 when constructing the physical vulnerability index.

Within the social capacity module, principal components PC 2, PC 4, PC 8, and PC 10 were selected. PC 2 captures communities characterized by a higher proportion of older residents, older housing stock, and limited access to plumbing. PC 8 is primarily associated with plumbing availability, while PC 10 is driven by lack of internet access. In the logistic regression, PC 8 received the largest positive coefficient among the selected components.

Residual spatial autocorrelation was weak across all three modules, with Moran's I values of 0.03 for the physical vulnerability (*p* = 0.02), hazard (*p* = 0.02), and social capacity (*p* = 0.03) models. These results suggest that any remaining spatial dependence is limited and unlikely to materially affect the substantive conclusions.

To assess the potential magnitude of selection bias, we conducted a descriptive screening in Section S6 in Supporting Information S1, comparing summary statistics of key high‐weight variables in the hybrid supervised–unsupervised framework between tested and untested regions. We did not observe meaningful differences for variables in the social capacity module, suggesting that testing patterns are only weakly related to socioeconomic conditions. Here, “meaningful differences” are operationalized as differences in means between tested and untested grid cells exceeding 0.25 standard deviations, where the standard deviation is calculated based on the tested grid cells. In contrast, differences were observed for selected variables in the hazard module, particularly those related to potential contamination sources, as well as for a few variables in the physical vulnerability module, including measures of soil properties, groundwater conditions, land development, and surface characteristics. These patterns suggest that selection bias may affect certain environmental and hydrogeologic predictors, although its overall impact on the composite risk score appears to be limited.

### 3.3 Spatial Distribution of Module Indices and Composite Risk Score

**Figure 4:**
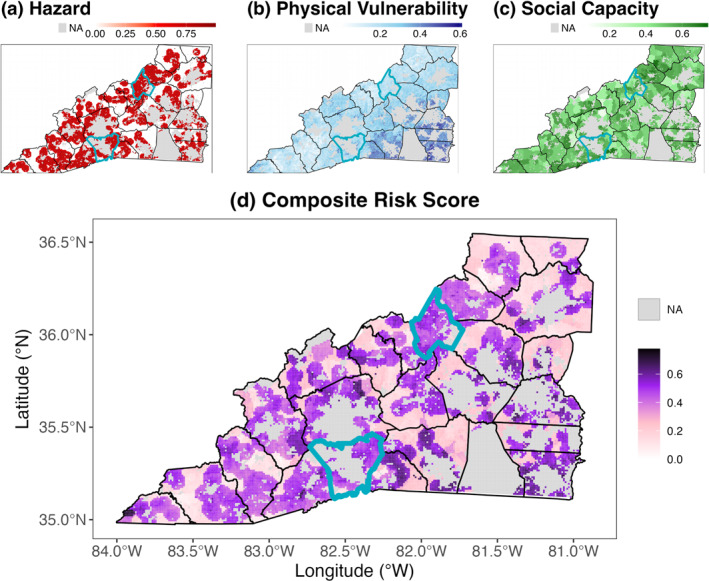
Spatial distribution of the hazard index, physical vulnerability index, social capacity index, and composite risk score across 25 counties in western North Carolina. Regions shown in gray indicate areas that do not rely on private wells. The boundaries of Avery and Henderson counties, where ground‐truthing was conducted, are emphasized with bold, teal outlines. Distinct color palettes are used for each panel to reflect the conceptual interpretation of each module: warmer tones (red) denote higher hazard, consistent with increased risk; cooler tones (blue) denote higher physical vulnerability, indicating weaker conditions; and green tones denote higher social capacity, reflecting greater resilience. These choices are intended to enhance interpretability within each module. As a result, color intensities are not directly comparable across panels.

Figure 4 illustrates the spatial distribution of the three module‐specific indices and the composite risk score across areas relying on private wells in the 25 western North Carolina counties most affected by Hurricane Helene. The hazard index exhibits strong spatial clustering, with elevated values concentrated along major river corridors and low‐lying basins, while upland and ridge regions show consistently lower values; summary statistics (Table 3; Table S9 in Supporting Information S1) support this pattern, and Figure S2 in Supporting Information S1 shows that the hazard index is highly right‐skewed, indicating that high hazard levels are concentrated in a relatively small number of locations. The physical vulnerability index displays a more diffuse but regionally coherent pattern, with higher values concentrated in the southern tier and eastern foothills and lower values in the northern mountain region; Figure S2 in Supporting Information S1 and its summary statistics (Table 3; Table S9 in Supporting Information S1) reflect a broader spread consistent with this gradient. The social capacity index shows moderate clustering, with higher‐capacity areas concentrated in parts of the northern and central counties and along the southern border, and lower‐capacity areas appearing more fragmented; corresponding summary measures (Table 3; Table S9 in Supporting Information S1) indicate less extreme variation relative to the other modules.

**Table 3:** Descriptive Statistics of Composite Risk Scores Over the 25 Western North Carolina Counties

County	Mean ± SD	Median [IQR]
Alexander	0.28 ± 0.13	0.23 [0.21–0.28]
Alleghany	0.23 ± 0.14	0.16 [0.15–0.19]
Ashe	0.25 ± 0.15	0.18 [0.13–0.42]
Avery	0.47 ± 0.09	0.49 [0.45–0.52]
Buncombe	0.44 ± 0.14	0.48 [0.36–0.53]
Burke	0.38 ± 0.16	0.33 [0.23–0.53]
Caldwell	0.37 ± 0.17	0.45 [0.18–0.50]
Catawba	0.47 ± 0.14	0.52 [0.34–0.56]
Clay	0.35 ± 0.18	0.42 [0.14–0.47]
Cleveland	0.43 ± 0.18	0.54 [0.25–0.58]
Gaston	0.51 ± 0.13	0.55 [0.52–0.59]
Haywood	0.38 ± 0.15	0.43 [0.28–0.48]
Henderson	0.44 ± 0.12	0.47 [0.44–0.51]
Jackson	0.34 ± 0.16	0.42 [0.15–0.46]
Lincoln	0.39 ± 0.15	0.49 [0.24–0.53]
Macon	0.32 ± 0.17	0.43 [0.14–0.48]
Madison	0.37 ± 0.15	0.39 [0.22–0.50]
McDowell	0.35 ± 0.16	0.45 [0.19–0.50]
Mitchell	0.34 ± 0.16	0.41 [0.18–0.49]
Polk	0.42 ± 0.18	0.51 [0.24–0.58]
Rutherford	0.35 ± 0.17	0.26 [0.21–0.54]
Transylvania	0.42 ± 0.13	0.46 [0.41–0.49]
Watauga	0.37 ± 0.13	0.43 [0.38–0.45]
Wilkes	0.29 ± 0.16	0.21 [0.16–0.47]
Yancey	0.32 ± 0.16	0.24 [0.19–0.48]

*Note.* The table summarizes the mean, standard deviation (SD), median, and interquartile range (25th to 75th percentile) of the composite risk score.

The composite risk score exhibited substantial spatial heterogeneity (Figure 4d), with elevated risk values forming irregular clusters that broadly coincided with areas of higher hazard and physical vulnerability. Higher‐risk zones were most prominent in the southern tier (Polk, Rutherford, and Gaston Counties) and in parts of the eastern foothills (Burke and Catawba Counties), with additional localized clusters in northern counties such as Watauga, Ashe, and Alleghany (Table 3).

### 3.4 Empirical Validation Against NC DHHS Well Testing Data

**Figure 5:**
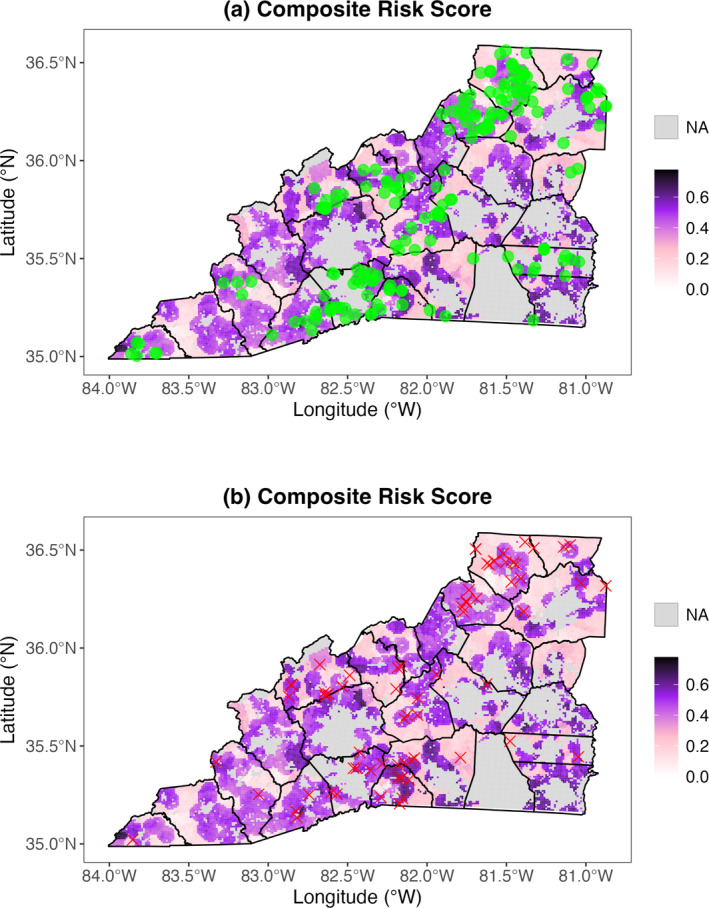
Spatial distribution of composite risk scores across western North Carolina, overlaid with total coliform well‐test results from the testing data set. Darker purple shading denotes higher risk. (a) Green circles represent uncontaminated wells. (b) Red crosses represent contaminated wells.

Within the testing subset of the NC DHHS well testing data (n=249), 176 wells tested negative and 73 tested positive for total coliform, corresponding to a contamination rate of 29.3%. Wells with positive test results were more frequently observed in areas with higher composite risk scores (Figure 5). A Wilcoxon rank‐sum test indicates that risk scores for contaminated wells are significantly higher than those for uncontaminated wells (p=0.006). This finding is robust to alternative formulations of the composite risk score; in a sensitivity analysis using a multiplicative specification (Hazard × Vulnerability × 1/Capacity), contaminated wells likewise exhibited significantly higher risk scores (*p* = 0.009). Details are provided in the Section S4 and Table S8 of Supporting Information S1.

### 3.5 Community Engagement and Qualitative Assessment

In Avery County, field observations documented variation in housing age, size, and condition, as well as evidence of infrastructure damage, erosion, and vegetation loss. Notably, two areas that appeared similarly vulnerable in the social capacity index (Figure 4) exhibited contrasting on‐the‐ground conditions. Homes in the northern area, located near a ski resort, were generally larger, newer, and in visibly better condition, whereas the southern area contained older, smaller homes, including many mobile homes, and showed more pronounced signs of deterioration. These discrepancies suggest that the smoothed, grid‐based index may mask important local variation within grid cells.

In Henderson County, the social capacity index displayed a wider range of values, producing more clearly delineated areas of elevated vulnerability. Windshield assessments identified corresponding variation in housing condition and infrastructure damage, which informed the selection of specific streets for outreach.

Conversations with local health department officials identified a subset of social capacity variables perceived as more directly indicative of household vulnerability and capacity to protect private well water. Based on this feedback, the initial set of 23 variables in the social capacity module was reduced to a final set of 13 (Table S1). Variables retained in the final set were those considered to have clearer connections to a household's ability to prevent, detect, or respond to well contamination during and after flooding events. In addition, discussions with residents along surveyed roads indicated limited awareness of free bacterial well testing programs and low rates of prior testing, further supporting the relevance of information access and engagement as components of social capacity.

## 4 Discussion

### 4.1 Interpretation of the Hybrid Framework and Risk Drivers

We developed a hybrid supervised–unsupervised framework to assess post‐hurricane private well contamination risk by integrating a large set of variables across hazard, physical vulnerability, and social capacity domains. Our hybrid supervised–unsupervised framework consistently outperformed both a fully supervised kitchen‐sink model and a fully unsupervised PCA‐based index, although overall differences in predictive performance were modest. We also found that the constructed social capacity index from this approach was aligned with NC DHHS monitoring data and community ground‐truthing.

These findings suggest that combining dimensionality reduction with outcome‐guided feature selection helps balance noise reduction and information retention. In particular, PCA mitigates redundancy among correlated covariates, while the supervised step ensures that retained components remain relevant for contamination risk, avoiding the information loss inherent in purely unsupervised summaries. The value of this approach is in a balance between theory‐driven approaches (such as the methods underpinning the CDC Social Vulnerability Index) and validation. This balance supports broader generalizability while reducing sensitivity to analytical choices such as variable selection and dimensionality reduction.

The selected principal components highlighted distinct environmental and social mechanisms shaping contamination risk. In the hazard module, the absence of the event‐specific flood inundation variable among selected components likely reflects limited spatial variability within the study area, which focused on counties most severely affected by Hurricane Helene. In contrast, components capturing broader flood exposure and wastewater‐related contamination sources (PCs 5 and 6) were influential, underscoring the combined importance of hydrologic context and infrastructure‐related risk factors in shaping post‐hurricane hazard conditions. In the physical vulnerability module, PC 1 emphasizes hydrogeologic settings that facilitate rapid infiltration and efficient subsurface transport, indicating that intrinsic groundwater susceptibility plays a dominant role in contamination risk (Shapiro & Falcone, 2022), while PC 10 reflects the contribution of coupled surface–groundwater pathways and subsurface storage (Xu et al., 2021). In the social capacity module, the selection of PC 2 highlights how demographic vulnerability and aging infrastructure can constrain effective response to contamination events (Li et al., 2023; Rehman et al., 2019), while the dominant weighting of PC 8 underscores the central role of plumbing availability and PC 10 points to the importance of information access in shaping post‐disaster response capacity.

Spatial patterns in the composite risk score reveal pronounced clustering in flood‐prone river corridors and low‐lying basins, reflecting the combined influence of flood exposure, hydrogeologic susceptibility, and social capacity. Several high‐risk areas align with known flood‐prone regions, suggesting that these processes jointly amplify contamination risk rather than acting in isolation. In contrast, lower‐risk areas are more commonly observed in higher‐elevation regions, where reduced flood exposure and intrinsic groundwater susceptibility likely mitigate overall risk. Together, these spatial patterns illustrate the value of the composite framework in capturing geographically heterogeneous contamination risk that may not be apparent when considering individual modules alone.

### 4.2 Validation and Community Ground‐Truthing

Validation using held‐out well testing data provides additional support for the framework. Wells with detected contamination tend to be located in areas assigned higher composite risk scores, indicating that the index captures spatial variation associated with contamination risk. While these results do not imply contamination prevalence among all wells in this study region, they suggest that the composite risk score is informative for distinguishing higher‐ and lower‐risk areas among tested wells.

Although the overall discriminative performance is modest (AUC ≈ 0.64), this should be interpreted in light of the intended use of the framework as a screening and prioritization tool rather than a predictive model for individual wells. In practice, the score is intended to rank areas by relative risk. Under this use, the key consideration is the tradeoff between false negatives (failing to prioritize high‐risk areas) and false positives (prioritizing lower‐risk areas). A modest AUC indicates that the score provides useful differentiation between higher‐ and lower‐risk areas and should be interpreted accordingly. Taken together, these results reinforce the potential value of the framework as a decision‐support tool for prioritizing well testing and outreach following extreme weather events.

Community engagement and qualitative assessment provide contextual insight into how the social capacity index relates to on‐the‐ground conditions and highlight important limitations of spatial indices, particularly in areas where similar index values may mask heterogeneous local conditions. In Avery County, vulnerable or spatially isolated households were not always apparent from roadway‐based observations despite contributing to elevated index values. In contrast, in Henderson County, clearer spatial gradients in index values facilitated more targeted outreach, underscoring the practical value of combining spatial indices with local knowledge and field verification. Feedback from local health officials further emphasizes the importance of balancing interpretability with comprehensiveness, as simplifying indices may improve usability but risks overlooking key dimensions of social capacity such as education, income, and access to information.

We note that these observations were not systematically coded into quantitative measures and therefore do not constitute formal statistical validation, but rather serve as a qualitative, field‐informed assessment that supports interpretation and refinement of the index.

### 4.3 Strengths

A central strength of this work is its hybrid supervised and unsupervised learning framework, which enables us to construct a risk score that is based on the theoretical framework of flood risk assessment and validated using empirical observations. The unsupervised component summarizes high‐dimensional information from the hazard, physical vulnerability, and social capacity modules, allowing the model to borrow strength across many correlated variables. The supervised component then uses labeled contamination outcomes to identify and weigh the most informative features. Together, these steps produce a single risk score that preserves informative structure in the data and remains effective in distinguishing contaminated wells from uncontaminated wells. Because this approach retains both an overall risk score and module‐specific indices, it offers complementary perspectives for prediction and interpretation: the composite score highlights regional patterns of elevated risk, while the module‐level indices clarify the underlying factors that contribute to those patterns. By using the LASSO penalty during index construction, the selected principal components within each module remain interpretable. The induced sparsity in the model coefficients allows us to identify a small subset of variables that contribute most strongly to explaining contamination risk.

Another strength of this study is its dual validation strategy. Statistical evaluation using held‐out testing data demonstrates that the risk score meaningfully differentiates contaminated from uncontaminated wells, while community ground‐truthing conducted by CWFNC confirms that the identified high‐risk areas correspond to on‐the‐ground observations. Together, these validation steps show that the risk score reflects actionable and scientifically grounded patterns in private well contamination risk.

### 4.4 Limitations and Future Directions

One limitation of this study is the size and nature of the empirical validation data set, which comprised 623 submitted samples and therefore does not represent population‐level private well contamination. The relatively small sample size limits the stability and generalizability of performance estimates. In addition, total coliform contamination following a hurricane is driven by localized and transient factors, such as well construction, maintenance, and short‐term hydrologic conditions, which are not fully captured by the available area‐level covariates. These considerations suggest that further improvements in predictive accuracy will likely require richer and more targeted data. In particular, incorporating well‐level characteristics, more detailed hydrologic measurements, and temporally resolved environmental variables could help better capture the processes driving contamination and improve model performance.

More broadly, the modest AUC observed in this study may reflect not only limitations of the validation data set, but also the intrinsic difficulty of predicting post‐hurricane total coliform contamination from area‐level geospatial covariates alone. Contamination outcomes are likely influenced by highly localized and transient processes, including well construction and maintenance, short‐term hydrologic conditions, and household‐level exposure pathways, many of which are not captured in the available predictors. As a result, the outcome may be only partially predictable from spatial covariates, even under improved sampling conditions.

Beyond these challenges, contamination risk following a hurricane is also inherently dynamic and evolves over time. While the proposed risk score captures spatial patterns associated with contamination risk, it does not explicitly account for temporal variation and should therefore be interpreted as a static assessment. Incorporating temporal dynamics is both reasonable and important in this context, as risk can change rapidly due to processes such as contaminant transport, dilution, and environmental recovery. However, the appropriate form through which time should be integrated into the risk framework, and how it should interact with spatial predictors, remains unclear and would require substantial methodological development beyond the scope of the current study. As such, the proposed risk score is best interpreted as reflecting conditions within an early post‐event window and is intended to support prioritization during the initial recovery phase.

Because testing was voluntary and opportunistic, case‐control labels reflect detected contamination conditional on testing rather than true population‐level prevalence. Unequal testing uptake may bias these labels if participation is correlated with socioeconomic status, awareness, access to resources, or perceived risk (Hayes et al., 2025; Wait et al., 2020). For example, households with lower social capacity may be less likely to test due to barriers such as limited outreach, transportation, or time; however, when testing does occur, it may be triggered by suspected contamination, potentially inflating observed positivity. Conversely, delayed testing after floodwaters recede or remediation occurs could bias observed positivity downward. Because the proposed risk score is intended to support risk guidance and prioritization rather than to estimate population‐level contamination prevalence, such selection is less consequential than it would be for prevalence estimation. Nevertheless, access to testing kits, proximity to sample drop‐off locations, and other determinants of testing participation likely influenced who was able to submit samples, and contamination events outside the testing window or in unsampled wells were not captured.

These competing mechanisms introduce uncertainty into the observed outcomes and may affect the supervised stage by altering which PCs appear most predictive and by shifting the estimated logistic‐regression coefficients and resulting index weights. While we do not explicitly correct for such bias, future work could apply inverse‐probability weighting or propensity‐to‐test modeling given additional data on well‐owner demographics, area‐level socioeconomic conditions, outreach intensity, or distance to testing locations.

These limitations in the testing data are paralleled by similar constraints in the community‐informed assessment component, as both rely on non‐random, participation‐driven processes that may not represent the broader population. The community‐informed assessment has several limitations. The fieldwork included a small number of households (*n* = 17) across two counties and was based on windshield surveys and informal conversations that were not recorded in a structured way. Therefore, this component does not provide formal statistical validation and cannot be used to confirm or refute the composite contamination risk score. In addition, outreach areas were selected based on low social capacity and input from local health officials, resulting in a targeted, non‐random sample that may not represent the broader study region. Future work could include more systematic field validation, such as structured surveys, larger and more representative samples, and improved integration of field observations with model outputs to enhance evaluation at finer spatial scales.

A further limitation is that the study does not explicitly evaluate the decision‐making utility of the proposed risk score under real‐world resource constraints. While the framework is intended to support prioritization of well testing and outreach, translating predictions into actions requires specifying tradeoffs between identifying high‐risk wells and allocating resources to lower‐risk locations. Formal decision‐analytic approaches, such as net benefit or decision curve analysis, could provide a more policy‐relevant assessment, but require context‐specific cost‐benefit assumptions that are not available here. In addition, the transferability of the framework has not been evaluated using external data sets from other hurricane events or regions, which remain limited. Future work could address these gaps through decision‐oriented evaluation and external validation to assess robustness and generalizability.

## 5 Conclusions

As the proportion of category 4 and 5 hurricanes is expected to increase due to climate change across the US Atlantic coast (Salarieh et al., 2023), this work provides a potential reference for NC DHHS and other state agencies to conduct rapid‐response sampling campaigns on microbial contamination after hurricane flooding. The combination of a composite risk score and module‐specific indices offers opportunities to visualize state‐level patterns in risk while maintaining an understanding of which areas are uniquely impacted by differences in environmental hazards, physical vulnerability, or social capacity. These approaches ultimately can help prioritize limited testing resources after a disaster occurs.

Our novel attempt to evaluate the performance of the risk score using quantitative well test data from NC DHHS and a qualitative community‐informed assessment also provides a strategy for future community‐engaged work to promote more equitable disaster response. We document not only the significance of validating that contamination risk scores can appropriately identify the most impacted communities using both qualitative and quantitative approaches, but also that public outreach and communication to promote standardized, routine well testing may be important climate adaptation and mitigation measures.

## Inclusion in Global Research Statement

This study incorporated community‐informed assessment to evaluate the social capacity module of the risk score. Clean Water for North Carolina collaborated with the research team to conduct fieldwork in Avery County and Henderson County, North Carolina. Their staff assisted with on‐the‐ground observations and evaluation of mapped risk patterns and coordinated engagement with local health department officials. This collaboration supported interpretation of spatial results and ensured that model outputs were evaluated in the context of local conditions and public health practice.

## Conflict of Interest

The authors declare no conflicts of interest relevant to this study.

## Supporting information

Supporting Information S1

Table S1

## Data Availability

The data used to generate the figures and tables in this manuscript and the supporting information, are publicly available through Harvard Dataverse (Lin, 2026). The code used to reproduce the figures and tables in this manuscript and the supporting information is publicly available on GitHub at https://github.com/water‐health‐opportunity‐lab/ncwell and archived on Zenodo (Lin et al., 2026). All data analyses and visualizations were conducted using *R* (R Core Team, 2020) based on the version 4.5.1.
