# Supplementary material for: Hybrid Supervised‐Unsupervised Modeling for Post‐Hurricane Private Well Contamination Risk Score Using Empirical Validation and Community‐Informed Assessment
Source: Geohealth. 2026 Jun 17;10(6):e2026GH001858. doi: 10.1029/2026GH001858 (PMC13273845; doi:10.1029/2026GH001858)
Supplement: Supplementary file 1 — Supporting Information S1 [file GH2-10-e2026GH001858-s002.pdf]

Supporting Information for "Hybrid supervised-unsupervised modeling for post-hurricane private well contamination risk score using empirical validation and community-informed assessment"

Jilei Lin[1,*], Jennifer Zhang[2,3], Ellen Wei[2], Kyndra Shea[2], Huixia Judy Wang[4], Tatiyana V. Apanasovich[1] Jahred M. Liddie[2], Erika Hernandez[5], Crystal Norford[5], Xindi C. Hu[2]

[1]Department of Statistics, The George Washington University, 801 22nd St NW, Washington, DC 20052, USA. ORCID: 0000-0002-7147-9851, 0009-0002-2416-9524

[2]Department of Environmental and Occupational Health, The George Washington University, 950 New Hampshire Ave NW #2, Washington, DC 20037, USA. ORCID: 0000-0002-4299-3931, 0009-0008-5437-1356, 0009-0005-9762-8420, 0009-0008-2375-5437, 0000-0003-2896-4015

[3]Thomas Jefferson High School for Science and Technology, 6560 Braddock Rd, Alexandria, VA 22312, USA. ORCID: 0009-0008-5437-1356

[4]Department of Statistics, Rice University, 6100 Main Street, Houston, TX 77005-1827, USA. ORCID: 0000-0002-5195-8564

[5]Clean Water for North Carolina, P.O. Box 69, Stem, NC 27581, USA.

*Corresponding author: Jilei Lin. Email: `jileil2@gwmail.gwu.edu`

Contents

1 Data Preprocessing **S3**

2 Summary statistics of variables **S3**

2.1 Hazard module S3

2.2 Physical vulnerability module S4

2.3 Social capacity module S6

3 Details of the hybrid supervised-unsupervised framework **S7**

3.1 Sign-constrained principal component analysis S7

3.2 Sensitivity analysis for the majority-voting threshold S9

3.3 Training and testing split and random forest S9

3.4 LASSO S10

3.5 Index construction S10

4 Sensitivity analysis for multiplicative composite-risk formulation S10

5 Summary statistics of module-specific indices S11

6 Selection Bias Screening S13

6.1 Numerical variables S15

6.2 Categorical variables S17

Section 1 describes the data preprocessing procedures applied prior to the PCA analysis. Section 2 summarizes the variables included in the PCA construction for the three modules (physical vulnerability, hazard, and social capacity). Section 3 provides additional details for the hybrid supervised-unsupervised framework. Section 4 presents a sensitivity analysis for the multiplicative composite-risk formulation. Section 5 summarizes the resulting module-specific indices across counties. Section 6 provides a descriptive screening of potential selection bias in the validation sample.

1 Data Preprocessing

We applied principal component analysis (PCA) to reduce dimensionality within each of the three modules. Because PCA is sensitive to outliers and highly correlated variables, we implemented a set of preprocessing steps prior to analysis. We first assessed the distribution of each variable and identified extreme values in both tails. Outliers were defined relative to the 1st and 99th percentiles of a fitted normal distribution. Transformation techniques (e.g., logarithmic, square-root, or power transformations) were applied when appropriate to reduce skewness. If transformations did not adequately mitigate the influence of extreme values, we replaced outliers with the corresponding 1st or 99th percentile values.

To further address multicollinearity, we evaluated the variance inflation factor (VIF) for continuous variables within each module. Following the conventional cutoff of 10, we iteratively removed variables with the highest VIFs until all remaining variables fell below this threshold. Finally, missing values were imputed using a k-nearest-neighbors (kNN) approach, where the five closest grid cells with non-missing values were used as reference. Continuous variables were imputed with the median, and categorical variables with the mode.

To address multicollinearity among categorical variables, we examined the generalized variance inflation factor (GVIF). This assessment was performed only for the vulnerability module, which contained the largest number of categorical predictors. Variable selection was guided by both subject-matter relevance and GVIF values. All retained categorical variables had GVIFs below 10, indicating acceptable multicollinearity levels.

2 Summary statistics of variables

In this section, we present the summary statistics of the variables in the hazard module, physical vulnerability module, and social capacity module. The detailed data dictionary for the variable description is presented in a separate Excel file `Table S1.xlsx`.

2.1 Hazard module

Table S2: Hazard module: (a) continuous variables with summary statistics.

Name	Mean	SD	Median	25%	75%	Unit
inundation_max	7.94	9.69	4.61	3.04	9.47	mm
sems_impact	0.00	0.01	0.00	0.00	0.00	unitless
cafo_impact	0.01	0.09	0.00	0.00	0.00	unitless
tri_water_releases	58.02	1454.99	0.00	0.00	0.00	lbs (annual)
tri_total_releases	2101.99	56973.28	0.00	0.00	0.00	lbs (annual)
owts	0.10	0.21	0.01	0.00	0.10	unitless
ag_runoff	55.50	2.73	55.95	55.49	56.00	lbs/acre

Table S3: Hazard module: (b) categorical variable with counts.

fema_floodplain	Count
FALSE	25261
TRUE	83

2.2 Physical vulnerability module

Table S4: Physical vulnerability module: (a) continuous variables with summary statistics.

Name	Mean	SD	Median	IQR	Unit
Pct_Wells	100.00	0.00	100.00	100–100	
dtw	12.76	6.51	11.75	8.41–15.80	m
trans	13.21	8.58	11.78	7.22–18.07	m^2/day
unsatTT	8.79	5.46	7.62	5.09–11.07	years
unsatWC	0.28	0.04	0.27	0.24–0.31	volumetric fraction
aq_rocktype	609.66	13.24	611.00	611–611	categorical
no3_dom	0.33	0.54	0.11	0.06–0.33	mg/L-N
no3_pub	0.24	0.39	0.09	0.05–0.23	mg/L-N
effrech	0.36	0.19	0.31	0.22–0.44	m/year
rech	430.50	175.48	375.94	309.22–504.59	mm/year
tmean	12.51	1.72	12.41	11.07–14.10	°C
ppt	1349.11	235.19	1282.92	1212.01–1422.62	mm
bfi	58.87	5.14	58.95	56.00–62.98	%
stm_den	2.37	1.21	1.74	1.52–2.82	km/km^2
ET	0.77	0.07	0.78	0.72–0.83	m/year
qf	0.23	0.06	0.21	0.19–0.26	m/year
rfor9045	73.20	12.75	75.00	65.89–81.57	%
PIMPV	1.51	1.95	0.97	0.44–1.74	%
PDEV	10.66	7.44	9.00	6.00–14.00	%

Continued on next page

Table S4 – continued from previous page

Name	Mean	SD	Median	IQR	Unit
PWETL95	0.02	0.04	0.01	0.00–0.02	%
PFOR	74.59	15.72	78.41	62.49–86.57	%
PFOR90	74.80	15.59	78.49	63.00–86.81	%
PAGP	10.99	9.62	8.50	3.04–16.14	%
Avg_Buff_Width	70.89	62.14	50.69	24.10–107.17	m
elevation	742.79	326.32	763.77	430.34–979.46	m
weighted_tfact	4.18	1.00	4.41	3.56–5.00	factor value
AWCmean	20.51	4.55	21.08	17.19–23.69	wt%
AWS25	3.51	0.37	3.51	3.27–3.77	cm
kfact	0.19	0.03	0.19	0.16–0.21	factor value
pH	5.17	0.20	5.19	5.06–5.30	pH
clay	19.32	5.62	17.63	15.01–21.97	wt%
om_kg_sq_m	18.16	8.63	16.62	11.91–22.13	kg/m^2
silt	25.57	4.04	25.09	22.81–27.70	wt%
soil_depth	159.09	31.18	163.61	135.03–184.12	cm
cec	7.69	3.05	6.75	5.51–9.43	cmol/kg
ec	0.00	0.00	0.00	0.00–0.00	dS/m
sar	0.00	0.02	0.00	0.00–0.00	ratio
db	1.45	0.04	1.45	1.42–1.48	g/cm^3

Table S5: Physical vulnerability module: (b) categorical variables with counts.

Category	Count
surfgeo	
Colluvial sediments, discontinuous	5811
Residual materials developed in igneous and metamorphic rocks	11286
others	1612
KB	
metamorphic rocks with unknown parent-rock ages	2787
paragneiss and schist precambrian metamorphic units	2903
late proterozoic (precambrian) metamorphic units	3343
others	9676
lith	
Biotite gneiss	3753
Gneiss	5110
others	9846
str_int	
Cryic	259
Frigid	66
Gelic	44

Continued on next page

Table S5 – continued from previous page

Category	Count
Hyperthermic	72
Isofrigid	79
Isohyperthermic	73
Isomesic	105
Isothermic	15571
Mesic	134
Pergelic	2306
Thermic	0
`hydgrp_int`	
A	2910
A/D	2389
B	12110
B/D	904
C	377
C/D	14
D	5
`landcover`	
Agriculture	2168
Barren Land	1
Developed	199
Forest	16229
Grassland	22
Shrubland	31
Water	32

2.3 Social capacity module

Table S6: Social capacity module: continuous variables with summary statistics.

Name	Mean	SD	Median	25%	75%	Unit
`pct_poverty`	24.15	8.81	22.80	18.12	29.64	%
`pct_women_gave_birth`	5.42	6.32	3.30	0.21	8.41	%
`pct_insured`	88.77	4.72	88.84	86.31	91.96	%
`pct_us_born`	96.12	2.97	96.46	94.85	98.22	%
`pct_no_move`	90.40	5.32	91.37	87.58	94.44	%
`pct_gov_workers`	15.58	6.44	15.52	10.52	20.47	%
`pct_minority`	8.54	8.98	6.00	2.72	11.23	%
`pct_limited_english`	0.65	2.31	0.00	0.00	0.00	%
`pct_single_parent`	8.19	9.38	6.10	1.12	11.35	%
`pct_unemployed`	4.43	5.82	2.57	0.00	6.38	%

Continued on next page

Table S6 – continued from previous page

Name	Mean	SD	Median	25%	75%	Unit
pct_no_college	11.80	7.95	10.83	5.84	16.13	%
pct_women	50.03	6.17	50.34	46.72	54.25	%
pct_over_65	27.66	9.71	27.28	20.60	34.08	%
pct_under_17	13.11	6.80	12.45	8.22	16.62	%
pct_owner_occupied	80.60	11.29	82.42	74.19	88.63	%
pct_active_commuting	1.73	3.34	0.00	0.00	2.13	%
pct_crowded_housing	2.07	3.97	0.00	0.00	2.79	%
pct_plumbing	96.33	4.89	97.65	94.42	100.00	%
pct_new_home	7.68	6.65	5.91	2.92	11.19	%
pct_no_vehicle	4.07	4.94	2.83	0.00	6.26	%
pct_no_internet	15.85	9.81	14.62	8.56	21.41	%

3 Details of the hybrid supervised-unsupervised framework

This section provides additional implementation details for the hybrid supervised–unsupervised framework. Section 3.1 describes the optimization problem and implementation of sign-constrained principal component analysis. Section 3.3 presents the training–testing split used for validation. Section 3.4 details the implementation of the logistic LASSO, including the specification of sign constraints and the selection of the penalization parameter. Finally, Section 3.5 describes the construction of the module-specific index, corresponding to Step 6 in Figure 2 of the main text.

3.1 Sign-constrained principal component analysis

Within a given module, let $\mathbf{X} \in \mathbb{R}^{n \times p}$ denote the module-specific data matrix, where n is the number of observations and p is the number of variables (columns). Each column of $\mathbf{X}$ was optionally centered and scaled to unit variance, yielding the standardized matrix $\tilde{\mathbf{X}}$. We then formed

$$\mathbf{S} = \frac{1}{n}\tilde{\mathbf{X}}^\top \tilde{\mathbf{X}},$$

which corresponds to the empirical correlation matrix when variables are standardized.

Let J_+ denote the index set of variables constrained to have nonnegative loadings and J_- the index set of variables constrained to have nonpositive loadings; all remaining variables were left unconstrained. The first constrained principal component loading vector $\mathbf{u}_1 \in \mathbb{R}^p$ was defined as the solution to

$$\max_{\mathbf{u} \in \mathbb{R}^p} \mathbf{u}^\top \mathbf{S}\mathbf{u} \quad \text{s.t.} \quad \|\mathbf{u}\|_2 = 1, \;\; u_j \geq 0 \; \forall j \in J_+, \;\; u_j \leq 0 \; \forall j \in J_-. \tag{1}$$

This formulation maximizes explained variance subject to partial sign constraints on the loading vector.

The optimization problem in (1) was approximately solved using a projected power-iteration scheme with multiple random starts. From an initial unit-norm vector $\mathbf{u}^{(0)}$, each iteration computed a power update $\mathbf{v} = \mathbf{S}\mathbf{u}^{(t)}$, enforced the sign constraints via elementwise projection ($v_j \leftarrow \max\{v_j, 0\}$ for $j \in J_+$ and $v_j \leftarrow \min\{v_j, 0\}$ for $j \in J_-$), and normalized the result to obtain the next iterate $\mathbf{u}^{(t+1)} = \mathbf{v}/\|\mathbf{v}\|_2$. Iterations were terminated when the objective value $\mathbf{u}^\top \mathbf{S}\mathbf{u}$ stabilized within a prescribed tolerance.

Additional constrained principal components were extracted sequentially by repeatedly applying the same constrained PCA procedure after accounting for the variance explained by previously identified components. This process was continued until the desired number of constrained principal components was obtained.

Let $\mathbf{U} = [\mathbf{u}_1, \ldots, \mathbf{u}_K]$ denote the resulting matrix of constrained principal component loadings. These constrained principal components were subsequently used in downstream modeling.

We also added a comparison of scree plots for ordinary PCA and sign-constrained PCA across modules. As shown in Figure S1, imposing sign constraints leads to some loss of explained variance, but the reduction is small. In particular, for the physical vulnerability module, the two scree curves nearly overlap.

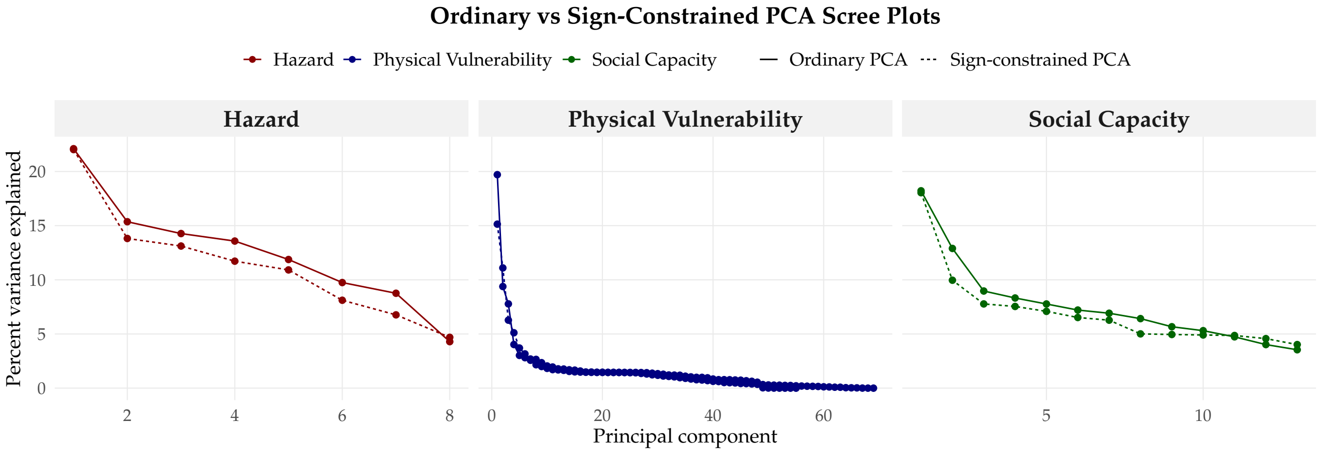

Figure S1. Comparison of scree plots for ordinary PCA and sign-constrained PCA across the hazard, physical vulnerability, and social capacity modules. The curves are generally similar across methods, indicating that the variance loss induced by sign constraints is modest. The overlap is especially close for the physical vulnerability module.

To maintain interpretability, we imposed sign constraints on the coefficient estimates in the module-specific logistic LASSO models: coefficients were constrained to be nonnegative for PCs from the physical vulnerability and hazard modules, and nonpositive for PCs from the social capacity module. The LASSO tuning parameter was selected by 10-fold cross-validation using the area under the ROC curve (AUC) as the performance criterion. We used $\lambda_{1\text{SE}}$ as the baseline sparse solution and then selected the value of λ along the cross-validated solution path whose model size, measured by the number of nonzero coefficients, was closest to a target of five active PCs per module. This procedure balances predictive performance, sparsity, and interpretability.

3.2 Sensitivity analysis for the majority-voting threshold

To reduce sensitivity to any single random split, we repeated the sign-constrained logistic LASSO procedure over 500 independent random splits and recorded how often each PC was selected within each module. Our main analysis retained PCs selected in more than 50% of runs. We chose this threshold as an intuitive stability criterion: a PC should be selected more often than not across repeated splits to be retained. A fully data-driven choice of the cutoff would require holding out additional data for validation, which we chose not to do given the limited sample size.

Table S7 summarizes how the selected PCs change when the voting cutoff is set to 40%, 50%, or 60%. The results show that the retained PCs are fairly stable across reasonable threshold choices. In particular, the hazard module consistently retains `haz_PC6` and `haz_PC5`, while the social capacity module consistently retains `cap_PC10`, `cap_PC8`, and `cap_PC2`. The physical vulnerability module shows somewhat greater sensitivity, but its top-ranked PCs remain `vul_PC10` and `vul_PC1`.

Table S7. Sensitivity of retained PCs to the majority-voting threshold across 500 repeated random splits.

Module	PCs selected at $> 40\%$	PCs selected at $> 50\%$	PCs selected at $> 60\%$
Physical Vulnerability	vul_PC10, vul_PC1, vul_PC9, vul_PC19	vul_PC10, vul_PC1	vul_PC10
Hazard	haz_PC6, haz_PC5	haz_PC6, haz_PC5	haz_PC6, haz_PC5
Social Capacity	cap_PC10, cap_PC8, cap_PC2, cap_PC4, cap_PC9	cap_PC10, cap_PC8, cap_PC2, cap_PC4	cap_PC10, cap_PC8, cap_PC2

3.3 Training and testing split and random forest

We split the validation dataset into training and testing subsets at a 3:2 ratio. Using the training data, we fit three random forest models that differ only in the features supplied: (1) the PCs selected in Step 1 of Figure 2, (2) all original variables from the three modules, and (3) the first PC from each module. Each model also includes the number of days since Hurricane Helene affected North Carolina. Hyperparameters, including the number of variables randomly sampled at each split and the minimum terminal node size, are tuned using five-fold cross-validation, and the optimal values are selected based on the highest cross-validated AUC. The decision threshold is then chosen to maximize the F1-score on the training data. This comparison allows us to evaluate how dimension reduction influences predictive performance and to assess the overall effectiveness of the PCA-based approach.

3.4 LASSO

To maintain interpretability, we impose sign constraints on the coefficient estimates: coefficients are constrained to be positive for PCs from the physical vulnerability and hazard modules, and negative for PCs from the social capacity module. We then select the LASSO tuning parameter using the standard one-standard-error (1SE) rule based on cross-validation, with an additional requirement that the resulting model size, measured by the number of nonzero coefficients, is as close as possible to a target of five active PCs per module. This combined criterion ensures that the selected model is both stable, due to the 1SE rule, and interpretable, due to the target model size.

3.5 Index construction

After obtaining the weights for each module, we aggregate the PCs using these weights to form the module-specific index. To enhance interpretability and approximate normality, we apply a power transformation guided by the Box–Cox transformation. The transformed index is then rescaled to the range 0–1 by subtracting its minimum value and dividing by its range. This rescaling procedure is performed separately for each module.

4 Sensitivity analysis for multiplicative composite-risk formulation

In the main analysis, we define the composite risk score additively as

$$\text{Risk} = \text{Physical Vulnerability} + \text{Hazard} - \text{Social Capacity}.$$

This choice yields a score that is numerically stable and directly interpretable after the module-specific indices are rescaled to the range 0–1. To address the reviewer's concern, we conducted a sensitivity analysis using the multiplicative form

$$\text{Risk}_{\text{mult}} = \text{Hazard} \times \text{Physical Vulnerability} \times \left(\frac{1}{\text{Social Capacity} + 0.001} \right).$$

The small constant avoids division by zero when the social capacity index is extremely close to zero.

This multiplicative form is numerically unstable when certain regions have very small social capacity values, because the reciprocal term can become very large and produce extreme raw risk scores. To make the resulting score interpretable, we standardized the multiplicative score to the range 0–1 using min–max scaling, as in the additive analysis. However, this transformation compresses most observations toward zero, so many regions receive very small standardized composite risk values. As a result, the multiplicative score is less practically useful for communication and interpretation, even if it remains statistically informative.

Table S8 summarizes the testing-set results for the multiplicative formulation. The mean standardized multiplicative risk score was 0.0006 in the detect group and 0.0005 in the non-detect group. Thus, the multiplicative score preserves the same ordering between the two groups but yields values that are numerically much smaller and less intuitive to interpret. Nevertheless, the Wilcoxon rank-sum test remained significant under the multiplicative formulation ($p = 0.0085$), indicating that the detect and non-detect groups are still distinguishable. The substantive conclusion therefore does not depend on whether the composite score is defined additively or multiplicatively.

Table S8. Sensitivity analysis for the multiplicative composite-risk formulation on the testing set.

Mean (detect)	Mean (non-detect)	Difference	Wilcoxon p
0.0006	0.0005	0.0001	0.0085

5 Summary statistics of module-specific indices

In this section, we summarize the distributions of the hazard, physical vulnerability, and social capacity indices. Figure S2 shows histograms of the three indices across the 25 western North Carolina counties. Table S9 reports county-level descriptive statistics for each index by module.

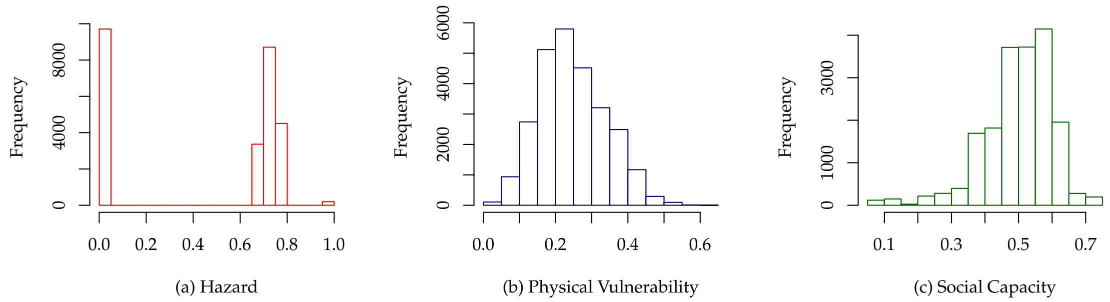

Figure S2. Histograms of the social capacity, hazard, and physical vulnerability indices for the 25 western North Carolina counties, restricted to areas where private well usage is 100%.

Table S9: County-wise descriptive statistics by module (Mean, SD, 25th percentile, Median, 75th percentile).

**County**	**Mean**	**SD**	**25%**	**Median**	**75%**
**(a) Hazard module**					
Alexander County	0.23	0.34	0.00	0.00	0.69

Continued on next page

Table S9 (continued)

County	Mean	SD	25%	Median	75%
Alleghany County	0.18	0.32	0.00	0.00	0.66
Ashe County	0.28	0.36	0.00	0.00	0.72
Avery County	0.64	0.26	0.71	0.74	0.76
Buncombe County	0.62	0.27	0.70	0.74	0.75
Burke County	0.47	0.34	0.00	0.70	0.73
Caldwell County	0.49	0.34	0.00	0.71	0.74
Catawba County	0.63	0.26	0.69	0.73	0.75
Clay County	0.43	0.35	0.00	0.69	0.72
Cleveland County	0.48	0.34	0.00	0.70	0.73
Gaston County	0.67	0.23	0.71	0.74	0.76
Haywood County	0.53	0.33	0.00	0.72	0.75
Henderson County	0.67	0.23	0.71	0.74	0.76
Jackson County	0.49	0.34	0.00	0.71	0.74
Lincoln County	0.46	0.35	0.00	0.69	0.73
Macon County	0.43	0.36	0.00	0.69	0.74
Madison County	0.34	0.36	0.00	0.00	0.71
Mcdowell County	0.41	0.36	0.00	0.68	0.73
Mitchell County	0.44	0.35	0.00	0.69	0.73
Polk County	0.46	0.35	0.00	0.70	0.74
Rutherford County	0.34	0.36	0.00	0.00	0.72
Transylvania County	0.62	0.28	0.69	0.74	0.76
Watauga County	0.59	0.30	0.68	0.73	0.76
Wilkes County	0.32	0.36	0.00	0.00	0.71
Yancey County	0.31	0.36	0.00	0.00	0.71
**(b) Physical vulnerability module**					
Alexander County	0.30	0.06	0.26	0.29	0.33
Alleghany County	0.19	0.04	0.17	0.19	0.21
Ashe County	0.16	0.04	0.13	0.16	0.19
Avery County	0.20	0.05	0.16	0.19	0.23
Buncombe County	0.23	0.05	0.19	0.22	0.25
Burke County	0.29	0.06	0.25	0.29	0.33
Caldwell County	0.27	0.06	0.23	0.26	0.30
Catawba County	0.34	0.08	0.27	0.34	0.40
Clay County	0.21	0.09	0.15	0.20	0.27
Cleveland County	0.36	0.04	0.33	0.36	0.38
Gaston County	0.41	0.06	0.37	0.40	0.44
Haywood County	0.17	0.07	0.13	0.17	0.22
Henderson County	0.24	0.06	0.20	0.24	0.28
Jackson County	0.14	0.06	0.10	0.13	0.17
Lincoln County	0.37	0.05	0.33	0.37	0.39
Macon County	0.15	0.05	0.11	0.15	0.19

Continued on next page

Table S9 (continued)

County	Mean	SD	25%	Median	75%
Madison County	0.25	0.05	0.22	0.26	0.29
Mcdowell County	0.27	0.05	0.24	0.27	0.31
Mitchell County	0.15	0.05	0.12	0.15	0.18
Polk County	0.33	0.08	0.26	0.35	0.39
Rutherford County	0.33	0.07	0.28	0.34	0.38
Transylvania County	0.21	0.06	0.17	0.21	0.25
Watauga County	0.20	0.05	0.16	0.19	0.23
Wilkes County	0.23	0.05	0.20	0.23	0.26
Yancey County	0.19	0.05	0.15	0.18	0.22
**(c) Social capacity module**					
Alexander County	0.45	0.09	0.35	0.48	0.51
Alleghany County	0.50	0.04	0.49	0.51	0.53
Ashe County	0.52	0.08	0.47	0.53	0.55
Avery County	0.49	0.10	0.38	0.50	0.59
Buncombe County	0.46	0.15	0.37	0.46	0.58
Burke County	0.46	0.07	0.43	0.43	0.52
Caldwell County	0.53	0.07	0.46	0.54	0.59
Catawba County	0.53	0.09	0.48	0.56	0.60
Clay County	0.51	0.14	0.56	0.57	0.58
Cleveland County	0.54	0.06	0.52	0.54	0.59
Gaston County	0.54	0.09	0.51	0.56	0.60
Haywood County	0.48	0.14	0.44	0.52	0.59
Henderson County	0.55	0.07	0.51	0.57	0.61
Jackson County	0.52	0.07	0.48	0.49	0.58
Lincoln County	0.55	0.06	0.51	0.55	0.60
Macon County	0.50	0.06	0.48	0.50	0.51
Madison County	0.44	0.16	0.40	0.46	0.56
Mcdowell County	0.52	0.07	0.49	0.53	0.56
Mitchell County	0.47	0.09	0.44	0.44	0.54
Polk County	0.49	0.11	0.39	0.45	0.54
Rutherford County	0.48	0.07	0.44	0.47	0.52
Transylvania County	0.55	0.07	0.52	0.55	0.60
Watauga County	0.60	0.04	0.57	0.58	0.64
Wilkes County	0.49	0.10	0.41	0.51	0.56
Yancey County	0.43	0.09	0.38	0.42	0.50

6 Selection Bias Screening

To assess the potential magnitude of selection bias in the validation dataset, we conducted a descriptive screening that compared tested and untested grid cells within the counties represented

by the testing dataset and restricted the analysis to cells with 100% private-well use. Rather than screening every candidate predictor, we focused on the variables most relevant to the hybrid supervised-unsupervised framework. Within each module, we retained the principal components that jointly explained at least 80% of the total variance, which yielded 6 retained PCs for the hazard module, 10 for the social capacity module, and 29 for the physical vulnerability module. We then identified high-weight variables as those with absolute PCA loadings greater than 0.25 on at least one retained PC and summarized their distributions separately for tested and untested grid cells.

This procedure yielded 8 high-weight hazard variables, 13 high-weight social-capacity variables, and 39 high-weight physical-vulnerability variables for descriptive screening. The retained high-weight hazard variables were `inundation_max`, `sems_impact`, `cafo_impact`, `tri_water_releases` `tri_total_releases`, `owts`, `ag_runoff`, and `fema_floodplain`. The retained high-weight social-capacity variables were `pct_poverty`, `pct_insured`, `pct_gov_workers`, `pct_minority`, `pct_limited_english`, `pct_unemployed`, `pct_no_college`, `pct_over_65`, `pct_owner_occupied`, `pct_plumbing`, `pct_new_home`, `pct_no_vehicle`, and `pct_no_internet`. The retained high-weight physical-vulnerability variables were `AWCmean`, `AWS25`, `bfi`, `cec`, `clay`, `db`, `dtw`, `effrech`, `elevation`, `hydgrp_int`, `KB`, `kfact`, `landcover`, `lith`, `no3_dom`, `no3_pub`, `om_kg_sq_m`, `PAGP`, `PDEV`, `PFOR`, `PFOR90`, `pH`, `PIMPV`, `ppt`, `PWETL95`, `qf`, `rech`, `rfor9045`, `sar`, `silt`, `soil_depth`, `stm_den`, `str_int`, `surfgeo`, `tmean`, `trans`, `unsatTT`, `unsatWC`, and `weighted_tfact`.

In the tables below, Q1 and Q3 denote the first and third quartiles, respectively. For numerical variables, a red '*' after the variable name indicates that the absolute difference between the untested mean and the tested mean exceeds 0.25 times the standard deviation of the tested group. We did not observe meaningful differences for the social-capacity variables, suggesting that testing patterns were only weakly related to socioeconomic conditions in this application. In contrast, differences were observed for selected hazard variables, particularly those related to potential contamination sources, and for several physical-vulnerability variables, including measures of soil properties, groundwater conditions, land development, and surface characteristics. These patterns suggest that selection bias may affect some environmental and hydrogeologic predictors, although its overall influence on the composite risk score appears to be limited.

6.1 Numerical variables

Table S10: Selection-bias screening for retained high-weight social-capacity variables. A red '*' after a variable name indicates that the absolute difference between the untested mean and tested mean exceeds 0.25 times the tested SD.

	Tested			Untested		
Variable	Mean	SD	Median [Q1, Q3]	Mean	SD	Median [Q1, Q3]
pct_poverty	23.91	8.83	21.74 [18.31, 29.21]	24.65	9.04	23.11 [18.48, 29.92]
pct_insured	88.94	5.13	88.99 [86.44, 93.07]	88.75	4.89	89.12 [86.22, 91.96]
pct_gov_workers	15.48	6.54	15.52 [10.11, 20.88]	15.54	6.47	15.46 [10.46, 20.12]
pct_minority	8.57	8.74	6.10 [3.09, 11.41]	8.46	9.28	5.75 [2.30, 11.23]
pct_limited_english	0.65	2.4	0.00 [0.00, 0.00]	0.63	2.25	0.00 [0.00, 0.00]
pct_unemployed	4.49	5.52	2.64 [0.00, 6.59]	4.62	5.86	2.75 [0.00, 6.49]
pct_no_college	11.17	8.14	10.13 [5.16, 15.52]	12.48	8.14	11.06 [6.77, 16.32]
pct_over_65	26.86	10.05	27.17 [19.30, 32.93]	27.42	9.68	27.44 [20.55, 33.96]
pct_owner_occupied	80.89	11.2	82.93 [75.11, 88.04]	80.91	11.28	82.90 [74.44, 88.84]
pct_plumbing	97.37	3.85	99.09 [95.90, 100.00]	96.47	4.75	97.93 [94.45, 100.00]
pct_new_home	8.15	7.01	5.94 [3.53, 11.99]	7.24	6.59	5.16 [2.52, 10.87]
pct_no_vehicle	4	5.06	2.54 [0.00, 5.90]	4.21	4.52	3.29 [0.00, 6.40]
pct_no_internet	14.55	9.75	13.96 [6.93, 21.05]	15.55	9.51	14.55 [8.56, 21.05]

Table S11: Selection-bias screening for retained high-weight hazard variables. A red '*' after a variable name indicates that the absolute difference between the untested mean and tested mean exceeds 0.25 times the tested SD.

	Tested			Untested		
Variable	Mean	SD	Median [Q1, Q3]	Mean	SD	Median [Q1, Q3]
inundation_max	2.55	1.06	2.25 [1.95, 3.11]	2.57	1.35	2.20 [1.81, 3.28]
sems_impact	0.00	0.02	0.00 [0.00, 0.00]	0.00	0.03	0.00 [0.00, 0.00]
cafo_impact	−3.81	0.73	−4.00 [−4.00, −4.00]	−3.74	0.84	−4.00 [−4.00, −4.00]
tri_water_releases	0.12	1.2	0.00 [0.00, 0.00]	0.23	3.11	0.00 [0.00, 0.00]
tri_total_releases	2.10	10.97	0.00 [0.00, 0.00]	3.64	27.6	0.00 [0.00, 0.00]
owts *	−4.22	3.39	−3.06 [−9.21, −1.48]	−5.66	3.45	−4.64 [−9.21, −2.41]

Continued on next page

Table S11: Selection-bias screening for retained high-weight hazard variables. A red '*' after a variable name indicates that the absolute difference between the untested mean and tested mean exceeds 0.25 times the tested SD. (continued)

	Tested			Untested		
Variable	Mean	SD	Median [Q1, Q3]	Mean	SD	Median [Q1, Q3]
ag_runoff *	55.01	2.31	55.54 [54.73, 55.93]	55.62	2.9	55.91 [55.47, 56.00]

Table S12: Selection-bias screening for retained high-weight physical-vulnerability variables. A red '*' after a variable name indicates that the absolute difference between the untested mean and tested mean exceeds 0.25 times the tested SD.

	Tested			Untested		
Variable	Mean	SD	Median [Q1, Q3]	Mean	SD	Median [Q1, Q3]
AWCmean *	21.8	4.45	22.36 [18.52, 25.11]	20.64	4.57	21.26 [17.20, 24.10]
AWS25 *	3.57	0.36	3.60 [3.32, 3.85]	3.47	0.35	3.46 [3.25, 3.70]
bfi	58.03	4.78	57.35 [55.02, 61.00]	58.43	5.17	58.11 [55.86, 62.22]
cec	6.86	2.02	6.27 [5.36, 8.38]	7.13	2.64	6.13 [5.40, 8.48]
clay	19.76	5.15	18.67 [15.72, 22.55]	20.13	6.23	18.30 [15.17, 23.48]
db	1.44	0.04	1.44 [1.41, 1.47]	1.44	0.04	1.44 [1.42, 1.47]
dtw *	10.64	5.96	9.72 [7.35, 12.53]	12.29	6.69	11.17 [7.88, 15.12]
effrech	0.33	0.15	0.31 [0.23, 0.41]	0.34	0.18	0.30 [0.21, 0.43]
elevation	689.72	263.91	695.63 [424.07, 897.35]	682.69	323.23	671.44 [381.39, 932.15]
kfact *	0.2	0.04	0.20 [0.18, 0.23]	0.19	0.04	0.19 [0.16, 0.21]
no3_dom	0.48	0.62	0.24 [0.09, 0.56]	0.35	0.56	0.12 [0.06, 0.36]
no3_pub	0.36	0.46	0.18 [0.07, 0.44]	0.25	0.41	0.09 [0.05, 0.26]
om_kg_sq_m	17.81	7.19	16.97 [12.23, 21.03]	16.95	8.35	15.24 [10.80, 20.78]
PAGP	13.14	8.82	11.15 [6.81, 18.27]	11.86	9.8	9.22 [3.31, 17.42]
PDEV *	12.81	7.11	11.00 [8.00, 15.00]	10.33	6.93	9.00 [6.00, 13.00]
PFOR	69.98	13.97	72.89 [59.36, 80.77]	73.13	16.03	77.28 [60.56, 85.81]
PFOR90	70.21	13.84	72.89 [59.51, 80.83]	73.37	15.9	77.31 [61.20, 85.91]
pH *	5.24	0.18	5.26 [5.13, 5.34]	5.18	0.2	5.21 [5.07, 5.31]
PIMPV	1.84	1.91	1.26 [0.68, 2.02]	1.49	1.87	1.00 [0.47, 1.68]
ppt	1297.26	184.67	1273.98 [1198.00, 1378.80]	1329.6	211.35	1273.60 [1207.81, 1380.18]

Continued on next page

Table S12: Selection-bias screening for retained high-weight physical-vulnerability variables. A red '*' after a variable name indicates that the absolute difference between the untested mean and tested mean exceeds 0.25 times the tested SD. (continued)

	Tested			Untested		
Variable	Mean	SD	Median [Q1, Q3]	Mean	SD	Median [Q1, Q3]
PWETL95	0.02	0.03	0.01 [0.00, 0.03]	0.02	0.04	0.01 [0.00, 0.03]
qf	0.21	0.05	0.20 [0.18, 0.23]	0.22	0.06	0.20 [0.18, 0.24]
rech	402.01	135.26	378.42 [326.11, 451.94]	400.03	162.75	361.72 [295.41, 446.92]
rfor9045 *	67.5	12.81	68.10 [56.47, 78.65]	73.82	11.33	75.00 [66.93, 81.57]
silt	25.65	3.96	25.28 [22.72, 27.73]	25.36	4.05	24.82 [22.55, 27.55]
soil_depth	166.4	31.6	174.33 [142.72, 191.06]	160.17	31.91	164.81 [134.88, 187.28]
stm_den	2.47	1.35	1.73 [1.52, 2.89]	2.25	1.11	1.70 [1.57, 2.66]
tmean	12.61	1.63	12.75 [11.08, 14.03]	12.73	1.76	12.81 [11.12, 14.34]
trans	16.23	12.47	15.06 [10.25, 20.41]	14.03	8.1	13.04 [7.89, 19.35]
unsatTT	8.02	5.41	6.60 [4.55, 9.68]	8.78	5.34	7.77 [5.25, 11.03]
unsatWC	0.29	0.04	0.29 [0.26, 0.31]	0.28	0.04	0.28 [0.25, 0.31]
weighted_tfact	4.32	0.99	5.00 [3.89, 5.00]	4.2	1.01	4.62 [3.56, 5.00]

6.2 Categorical variables

Table S13: Selection-bias screening for retained high-weight categorical hazard variables.

		Tested	Untested
Variable	Level	*n* (%)	*n* (%)
fema_floodplain	FALSE	516 (99.2%)	15279 (99.7%)
	TRUE	4 (0.8%)	45 (0.3%)

Table S14: Selection-bias screening for retained high-weight categorical physical-vulnerability variables.

		Tested	Untested
Variable	Level	n (%)	n (%)
`hydgrp_int`	1	63 (12.1%)	2111 (13.8%)
	2	72 (13.8%)	1670 (10.9%)
	3	351 (67.5%)	10284 (67.1%)
	4	28 (5.4%)	877 (5.7%)
	5	5 (1.0%)	369 (2.4%)
	6	1 (0.2%)	11 (0.1%)
	7	0 (0.0%)	2 (0.0%)
`KB`	mm1	70 (13.5%)	2597 (16.9%)
	others	450 (86.5%)	12727 (83.1%)
`landcover`	Agriculture	86 (16.5%)	1974 (12.9%)
	Barren Land	0 (0.0%)	1 (0.0%)
	Developed	23 (4.4%)	132 (0.9%)
	Forest	410 (78.8%)	13143 (85.8%)
	Grassland	0 (0.0%)	19 (0.1%)
	Shrubland	0 (0.0%)	30 (0.2%)
	Water	1 (0.2%)	25 (0.2%)
`lith`	others	520 (100.0%)	15324 (100.0%)
`sar`	0	520 (100.0%)	14848 (96.9%)
	1	0 (0.0%)	476 (3.1%)
`str_int`	10	8 (1.5%)	115 (0.8%)
	11	63 (12.1%)	1936 (12.6%)
	2	0 (0.0%)	115 (0.8%)
	3	0 (0.0%)	37 (0.2%)
	4	1 (0.2%)	28 (0.2%)
	5	0 (0.0%)	40 (0.3%)
	6	0 (0.0%)	43 (0.3%)
	7	3 (0.6%)	43 (0.3%)
	8	1 (0.2%)	62 (0.4%)
	9	444 (85.4%)	12905 (84.2%)

Continued on next page

Table S14: Selection-bias screening for retained high-weight categorical physical-vulnerability variables. (continued)

Variable	Level	Tested	Untested
		n (%)	n (%)
`surfgeo`	others	126 (24.2%)	5130 (33.5%)
	Residual materials developed in igneous and metamorphic rocks	394 (75.8%)	10194 (66.5%)
